# Intrahepatic microbes govern liver immunity by programming NKT cells

**DOI:** 10.1172/JCI151725

**Published:** 2022-04-15

**Authors:** Joshua C. Leinwand, Bidisha Paul, Ruonan Chen, Fangxi Xu, Maria A. Sierra, Madan M. Paluru, Sumant Nanduri, Carolina G. Alcantara, Sorin A.A. Shadaloey, Fan Yang, Salma A. Adam, Qianhao Li, Michelle Bandel, Inderdeep Gakhal, Lara Appiah, Yuqi Guo, Mridula Vardhan, Zia Flaminio, Emilie R. Grodman, Ari Mermelstein, Wei Wang, Brian Diskin, Berk Aykut, Mohammad Khan, Gregor Werba, Smruti Pushalkar, Mia McKinstry, Zachary Kluger, Jaimie J. Park, Brandon Hsieh, Kristen Dancel-Manning, Feng-Xia Liang, James S. Park, Anjana Saxena, Xin Li, Neil D. Theise, Deepak Saxena, George Miller

**Affiliations:** 1 S.A. Localio Laboratory, Department of Surgery, New York University (NYU) Langone Health, New York, New York, USA.; 2 Department of Molecular Pathobiology, NYU College of Dentistry, New York, New York, USA.; 3 Department of Pathology,; 4 Department of Medicine,; 5 Ronald O. Perelman Department of Dermatology, and; 6 Department of Cell Biology, NYU Langone Health, New York, New York, USA.; 7 Biology Department, Brooklyn College and Biology/Biochemistry Programs, Graduate Center (CUNY), New York, New York, USA.

**Keywords:** Hepatology, Microbiology, Antigen, Chemokines, Innate immunity

## Abstract

The gut microbiome shapes local and systemic immunity. The liver is presumed to be a protected sterile site. As such, a hepatic microbiome has not been examined. Here, we showed a liver microbiome in mice and humans that is distinct from that of the gut and is enriched in Proteobacteria. It undergoes dynamic alterations with age and is influenced by the environment and host physiology. Fecal microbial transfer experiments revealed that the liver microbiome is populated from the gut in a highly selective manner. Hepatic immunity is dependent on the microbiome, specifically the *bacteroidetes* species. Targeting *bacteroidetes* with oral antibiotics reduced hepatic immune cells by approximately 90%, prevented antigen-presenting cell (APC) maturation, and mitigated adaptive immunity. Mechanistically, our findings are consistent with presentation of *bacteroidetes*-derived glycosphingolipids to NKT cells promoting CCL5 signaling, which drives hepatic leukocyte expansion and activation, among other possible host-microbe interactions. Collectively, we reveal a microbial/glycosphingolipid/NKT/CCL5 axis that underlies hepatic immunity.

## Graphical abstract

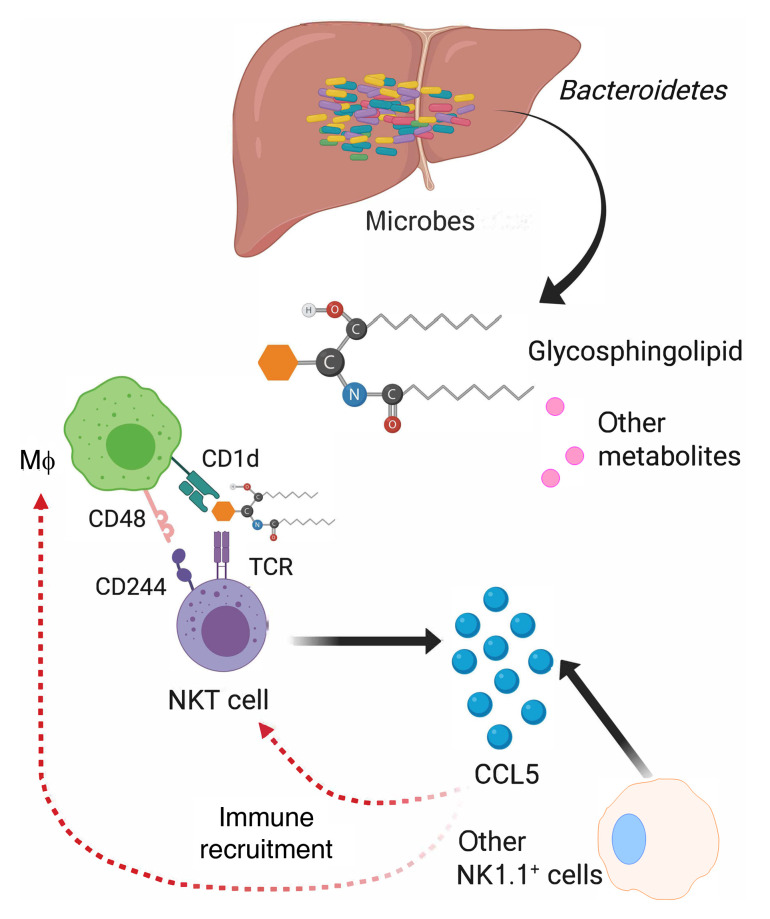

## Introduction

Gut microbiota represent the most abundant and diverse collection of commensal organisms in the body (1). The intestinal microbiome plays a fundamental role in shaping the host immune system (2). Interactions between commensal microbes and the host intestinal mucosa are necessary for the induction, training, and homeostatic functions of gut immunity; conversely, dysbiosis in the gut is associated with local barrier dysfunction, autoimmunity, and oncogenesis (3). The liver, much like the gut, is densely populated by a myriad of innate and adaptive immune cells that are critical to physiologic homeostasis. The liver serves as a selective barrier between the host and the external environment. As portal blood transports an array of foreign, but harmless, dietary and environmental antigens, the liver’s baseline status is immunotolerant and antiinflammatory; however, in select circumstances, including after acute hepatic injury, the liver is capable of mounting potent immunogenic responses (4, 5). The balance between hepatic tolerance and immunity is essential for organismal survival.

Whereas the intestinal microbiome directs immunity in the gut and beyond, the liver microbiome has not been characterized. In fact, the liver is generally regarded as a sterile organ. Consequently, the relationship between liver microbes and hepatic immunity is unexplored. We recently showed that the pancreas, which like the liver has a connection to the gastrointestinal tract via the ampulla of Vater, has a resident microbiome that governs intrapancreatic immunity (6). We postulated that the liver similarly harbors microbes that regulate hepatic immune function.

Here, we report the presence of a liver microbiome that is distinct from that of the gut and characterize its dynamic alterations under changing physiologic conditions. We identify specific bacterial taxa and predict their metabolic byproducts that potentiate liver inflammatory cell recruitment and maturation via NKT cell activation. Collectively, our data suggest that liver microbiota are critical in regulating the balance between hepatic immunity and tolerance and provide a rationale for microbial-based therapies in the treatment of liver disease.

## Results

### The liver harbors a microbiome.

**Figure 1:**
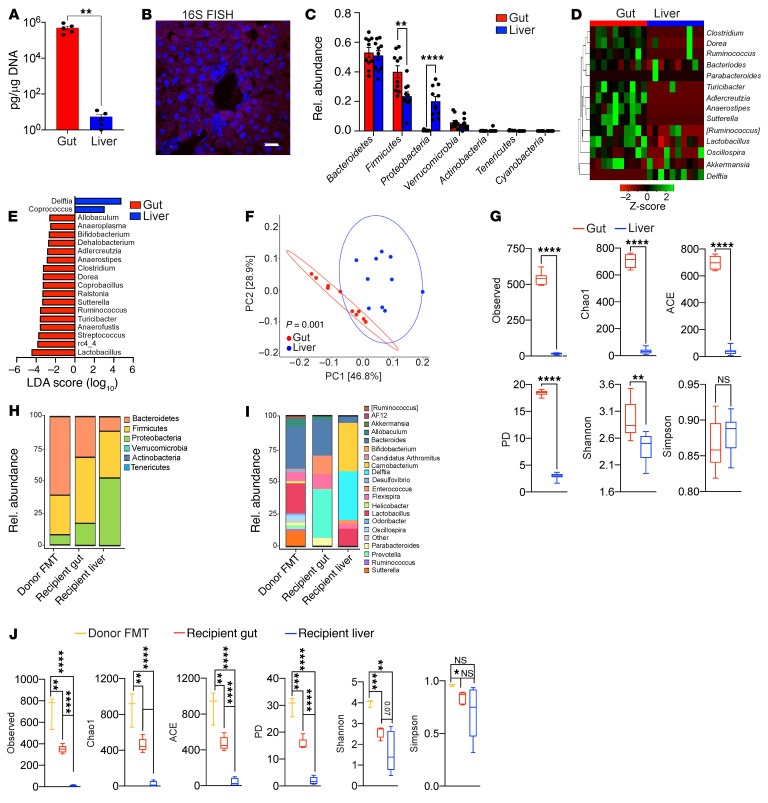
The hepatic microbiome is distinct from that of the gut in mice. (**A**) Bacterial DNA content was measured in gut and liver of 6-week-old female WT mice using qPCR. *n* = 5. **P < 0.01. (**B**) The presence of an intrahepatic microbiome was evaluated by FISH using a 16S probe. Representative images are shown. Scale bar: 20 μm. (**C**) Taxonomic composition of microbiota assigned to the phylum level in the gut and liver based on average percentage of relative (Rel.) abundance determined by 16S rRNA-Seq. *n* = 10. **P < 0.01; ****P < 0.0001. (**D**) Heatmap showing log_2_-transformed relative abundance of the most highly represented bacterial genera in liver and gut. (**E**) Linear discriminant analysis (LEfSe) based on 16S rRNA-Seq identified differentially abundant genera in liver (blue bars) and gut (red bars). (**F**) Weighted PCoA plots based on Bray-Curtis dissimilarity matrix. Each symbol represents a sample from liver (blue) or gut (red). Clusters were determined by pairwise PERMANOVA. The *x* and *y* axes indicate percentage of variation, and ellipses indicate 95% CI. (**G**) The liver and gut microbiomes in 6-week-old female WT mice were analyzed for α-diversity measures including observed OTUs, Chao1, ACE, PD, and Shannon and Simpson indices. **P < 0.01; ****P < 0.0001. (**H**–**J**) Germ-free mice were treated with FMT. Taxonomic composition of microbiota in the donor FMT slurry, recipient liver, and recipient gut were assigned to phylum (**H**) and genus (**I**) levels, and α-diversity measures were determined (**J**) based on 16S rRNA-Seq. *n* = 5. *P < 0.05; **P < 0.01; ***P < 0.001; ****P < 0.0001.

To determine whether the liver hosts a microbiome, we performed quantitative PCR (qPCR) using 16S primers on normal mouse liver tissues. A microbial population was detected in the liver, albeit at low abundance compared with that in gut; liver microbial abundance was readily distinguishable from that in reagent-only controls (Figure 1A, Supplemental Figure 1A, and Supplemental Table 1; supplemental material available online with this article; https://doi.org/10.1172/JCI151725DS1). Similarly, 16S FISH and transmission electron microscopy (TEM) revealed the presence of hepatic bacteria in the sinusoidal space as well as engulfed by Kupffer cells (Figure 1B and Supplemental Figure 1, B and C). Germ-free mice had no detectable liver microbiome (Supplemental Figure 1B). Of note, we successfully cultured bacteria from sterilely harvested liver tissue under both aerobic and anaerobic conditions (Supplemental Figure 1D). To characterize the liver microbiome, we performed 16S rRNA-Seq in 6-week-old female mice. Five distinct phyla were detected in the liver, with Bacteroidetes (~50%), Firmicutes (~25%), Proteobacteria (~20%), and Verrucomicrobia (~4%) being most abundant (Figure 1C). Of note, Proteobacteria represented an approximately 40-fold greater fraction of the liver microbiome compared with that of gut, whereas Firmicutes were approximately 2-fold more abundant in the gut. At the genus level, *Delftia* and *Coprococcus* were significantly expanded in the liver, whereas numerous taxa, including *Lactobacillus*, *Ruminococcus*, and *Clostridium*, were more prevalent in the gut (Figure 1, D and E). Assessment of clade abundances using linear discriminant analysis effect size (LEfSe) revealed differences in microbial composition between liver and gut across the entire taxonomic hierarchy (Supplemental Figure 1E). Principal coordinate analysis (PCoA) computed using weighted UniFrac distance metrics confirmed distinct microbial communities in liver versus gut (Figure 1F). α-Diversity measures indicated that the liver microbiome exhibited markedly decreased richness compared with that of the gut based on observed operational taxonomic units (OTUs), Chao1, the abundance-based coverage estimator (ACE), and phylogenic diversity (PD) and decreased evenness based on the Shannon index (6) (Figure 1G). Collectively, these data reveal that the normal mouse liver harbors a microbiome that is distinct from that of the gut.

### The hepatic microbiome is selectively populated from the gut.

We postulated that the source of liver bacteria is the gut microbiome. To test this hypothesis, we orally administered fluorescently labeled *Porphyromonas* *gingivalis*, based on its ability to translocate from the alimentary tract (7), and assessed its abundance in both the gut and liver. *P*. *gingivalis* translocated to the liver as predicted, and linear regression analysis indicated that its abundance remained stable in the liver over time despite temporal decreases in the stomach and duodenum (Supplemental Figure 1F). To determine whether selective pressure occurs as gut bacteria populate the liver, we administered fecal microbial transplants (FMTs) from specific pathogen–free (SPF) donor mice to germ-free mice. FMTs to germ-free mice successfully populated the liver alongside the gut (Figure 1, H and I). Moreover, whereas the composition of the recipient gut microbiome resembled that of the donor fecal microbiome, the liver microbiome was uniquely enriched for the phylum Proteobacteria, including the *Delftia* taxon. This recapitulated their increased abundance in the native hepatic microbiome (Figure 1, C–E, and Supplemental Figure 1E). Similarly, α-diversity was substantially reduced in the recipient liver compared with in the gut or donor FMT slurry (Figure 1J). Collectively, these FMT data indicate that the liver microbiome is selectively populated from the gut. Consistent with this notion, the microbiome in the central liver clustered more closely with the gut microbiome (Supplemental Figure 1G). Of note, the liver microbiome clustered most closely with the pancreatic microbiome compared with that of the duodenum or gallbladder, perhaps relating to their similar respective tissue host environments; *Lactobacillus*, *Akkermansia*, *Turicibacter*, and *Porphyromonas* were among the most prominent genera across all compartments tested (Supplemental Figure 1, H and I).

### The human liver microbiome is distinct from that of the gut.

**Figure 2:**
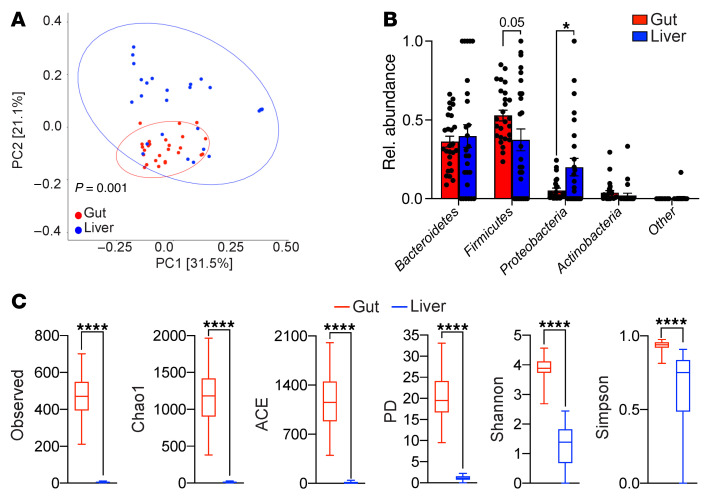
The hepatic microbiome is distinct from that of the gut in humans. (**A**–**C**) Matched liver and fecal specimens from 26 patients were analyzed by 16S rRNA-Seq. Weighted PCoA plots based on the Bray-Curtis dissimilarity matrix (**A**), the taxonomic composition of microbiota assigned to phylum level based on average percentage of relative abundance (**B**), and α-diversity measures (**C**) are shown. *P < 0.05; ****P < 0.0001.

To determine whether the human liver hosts a microbiome and whether it is distinct from that of the gut, we collected liver biopsies in a sterile manner and matched fecal specimens for microbial analysis from 26 patients undergoing hepatic resection (Supplemental Tables 1 and 2). Nearly all subjects had normal baseline liver function. In patients with liver tumors, hepatic biopsies were obtained a minimal distance of 3 cm away from the nearest tumor nodule. Consistent with our mouse data, the human liver harbored a microbiome that was much less abundant compared with that of the gut and that was distinguishable from reagent-only controls based on qPCR using 16S primers (Supplemental Figure 1, J and K, and Supplemental Table 1), and the human microbiome clustered separately from the gut on β-diversity analysis of 16S rRNA-Seq data (Figure 2A). Moreover, akin to our findings in mice, Proteobacteria was markedly more abundant in liver compared with gut, whereas Firmicutes was less abundant (Figure 2B). Likewise, α-diversity analyses revealed distinctly lower microbial richness in the human liver compared with gut based on OTU, Chao1, ACE, and PD, and decreased evenness based on the Shannon and Simpson indices (6), which again parallels our findings in mice (Figure 2C). Taken together, these data indicate that the human liver harbors a microbiome that is rich in Proteobacteria and distinct from that of the gut.

### The liver microbiome varies with age, sex, and environment.

**Figure 3:**
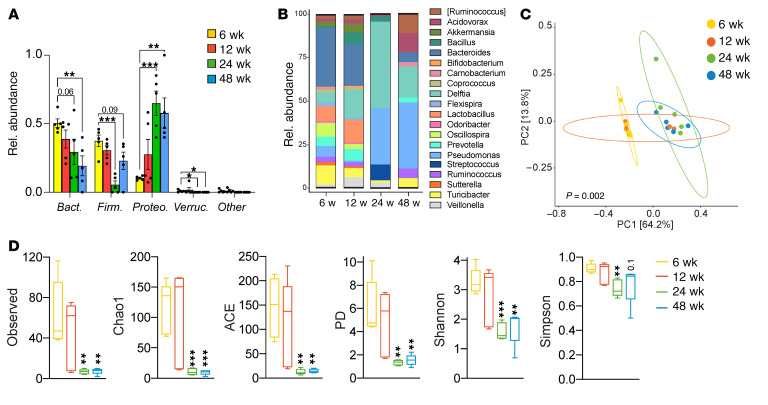
The liver microbiome varies with age. (**A**–**D**) We comparatively analyzed the hepatic microbiome in female mice aged 6, 12, 24, and 48 weeks by 16S rRNA-Seq. Taxonomic composition of microbiota in the liver were assigned to phylum (**A**) and genus (**B**) levels based on average percentage of relative abundance. Weighted PCoA plots from each cohort were based on the Bray-Curtis dissimilarity matrix (**C**), and changes in α-diversity measures compared with 6 weeks (**D**) are shown. *n* = 5/group. *P < 0.05; **P < 0.01; ***P < 0.001. Bact., Bacteroidetes; Firm., Firmicutes; Proteo., Proteobacteria; Verruc., Verrucomicrobia.

To determine how the liver microbiome changes with age, we comparatively analyzed the hepatic microbiome in female mice aged 6 to 48 weeks. Whereas the most abundant phyla in the liver of 6-week-old mice were Bacteroidetes and Firmicutes, as mice aged, these decreased and Proteobacteria became most abundant (Figure 3A). In contrast, Bacteroidetes and Firmicutes remained the most abundant phyla in the gut at all ages (Supplemental Figure 2, A and B). At the genus level, *Pseudomonas* and *Delftia* became the dominant taxa in the liver as mice aged (Figure 3B). PCoA showed distinct clustering of the hepatic microbial communities at different developmental ages in mice (Figure 3C). Further, as animals aged, richness and evenness markedly decreased in liver microbial communities (Figure 3D).

**Figure 4:**
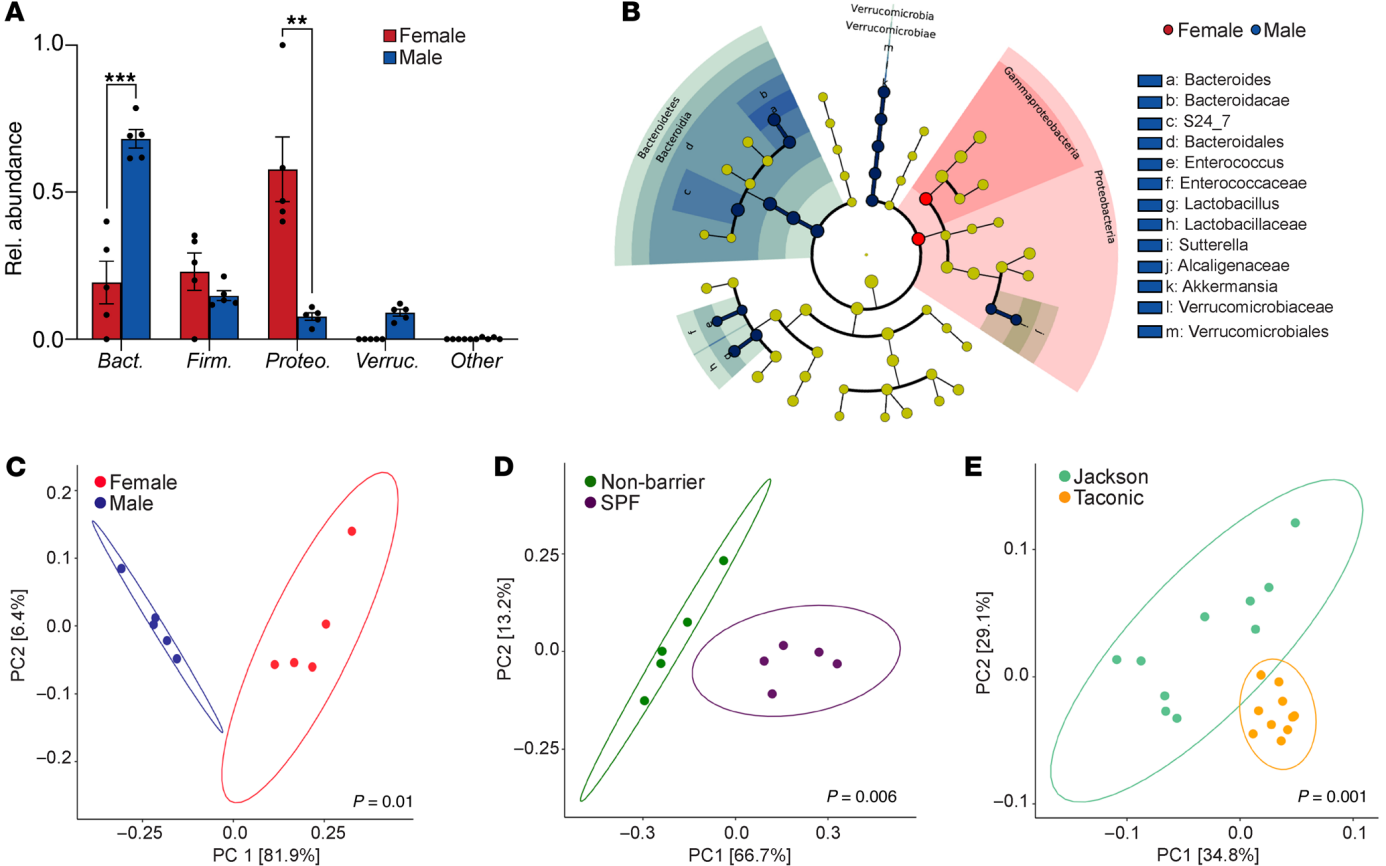
The liver microbiome varies with sex and environment. (**A**–**C**) We comparatively analyzed the hepatic microbiome in 48-week-old male and female mice by 16S rRNA-Seq. Taxonomic composition of microbiota in the liver were assigned to phylum level based on average percentage of relative abundance (**A**). Sex-based differences in the liver microbiome across all taxonomic hierarchies were detected by LEfSe and are shown in a cladogram (**B**). Weighted PCoA plots from each sex-based cohort (**C**). *n* = 5/group. **P < 0.01; ***P < 0.001. (**D**) Weighted PCoA plots of hepatic microbial communities from cohorts of female mice obtained at 6 weeks of age from The Jackson Laboratory and housed for 3 weeks in SPF or nonbarrier vivaria. *n* = 5/group. (**E**) Weighted PCoA plots of hepatic microbial communities from cohorts of 6-week-old female mice obtained from The Jackson Laboratory or Taconic Biosciences. *n* = 10/group.

We next assessed sex-specific variations in the liver microbiome by comparing adult (48 week old) female and male mice. Whereas adult females exhibited a predominance of Proteobacteria as shown above (Figure 3A), the most abundant phylum in adult males was Bacteroidetes (Figure 4A). Both female and male gut microbiomes had low abundances of Proteobacteria; the female gut microbiome was dominated by Bacteroidetes, while males had a higher abundance of Firmicutes (Supplemental Figure 2, C and D). Multiple additional differences were evident between the adult male and female liver microbiomes across the entire taxonomic hierarchy (Figure 4B). PCoA confirmed distinct hepatic microbial communities between females and males (Figure 4C). Further, the liver microbiome in males exhibited increased richness and evenness, which was less conspicuous in the gut microbiome (Supplemental Figure 2, E and F).

Since an organism’s environment can influence its resident microbiome (8), we compared the hepatic microbiomes of mice housed in SPF and nonbarrier facilities. Mice purchased from the same vendor developed distinct hepatic microbiomes by 3 weeks after transfer to SPF barrier versus nonbarrier vivaria (Figure 4D). The liver microbiome in each group was also distinct based on α-diversity analyses with mice in SPF barrier facilities, exhibiting increased richness and evenness (Supplemental Figure 2G). Similarly, mice purchased from different vendors, reported to have distinct gut microbes (9), also exhibited distinct liver microbiomes (Figure 4E). Taken together, these data indicate that the liver microbiome is dynamic and varies considerably in healthy hosts depending on age, sex, and environmental exposure.

### Modulating the gut microbiome alters bacterial communities in the liver.

**Figure 5:**
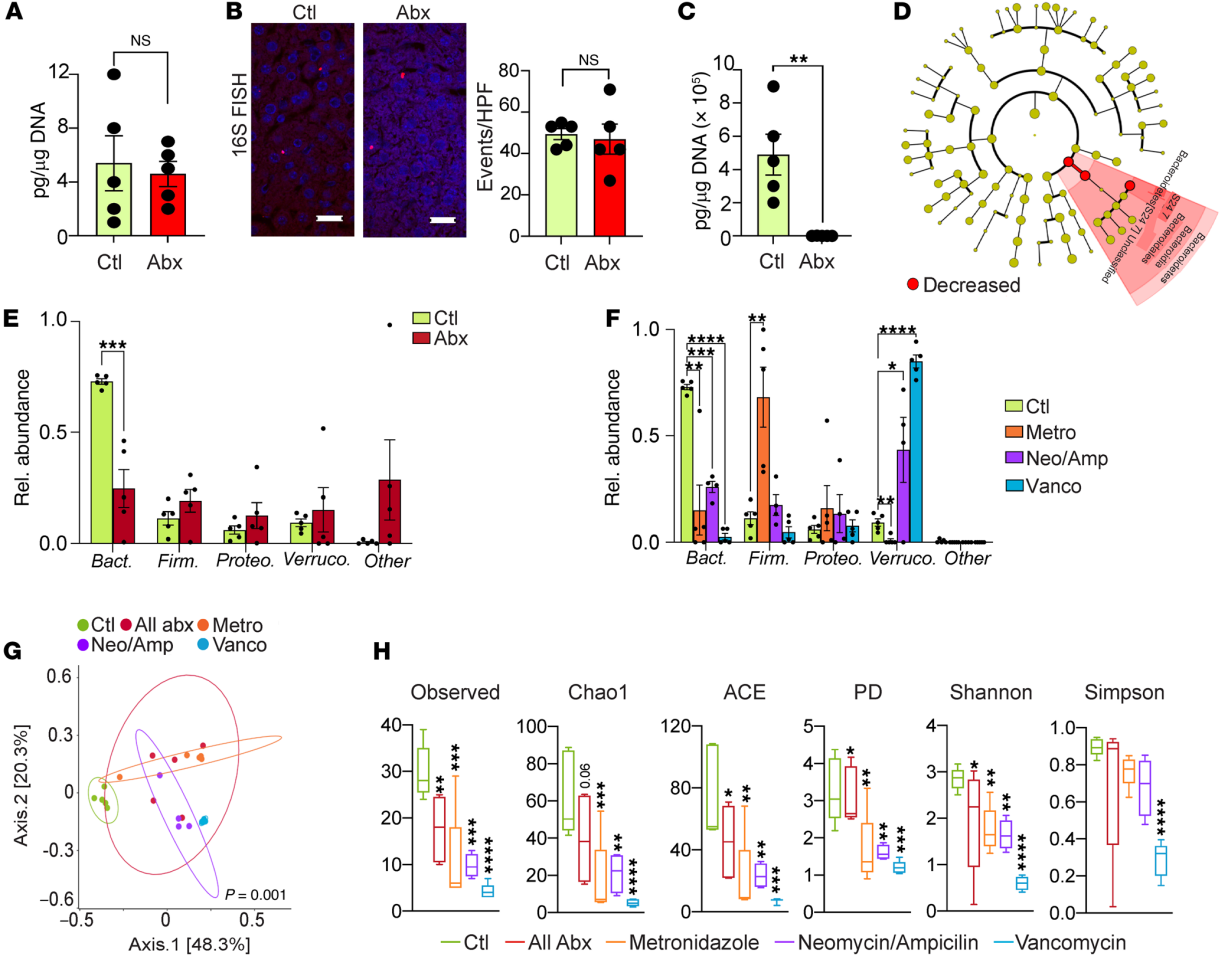
Antibiotic administration selectively modulates the bacterial composition of the liver microbiome. (**A**) Hepatic bacterial DNA content was comparatively analyzed by qPCR in cohorts of 6-week-old female mice treated with broad-spectrum antibiotics or vehicle. *n* = 5/group. (**B**) The intrahepatic microbiome was compared in the liver of mice treated with broad-spectrum antibiotics (Abx) or vehicle (Ctl) by 16S FISH. Representative images and quantitative results are shown. *n* = 5/group. Scale bars: 20 μm. (**C**) Gut bacterial DNA content was comparatively analyzed by qPCR in cohorts of 6-week-old female mice treated with broad-spectrum antibiotics or vehicle. *n* = 5/group. **P < 0.01. (**D**) Cladogram showing changes in abundance of microbes across the entire taxonomic hierarchy in the liver of mice treated with broad-spectrum antibiotics. (**E**) Taxonomic composition of microbiota assigned to phylum level in the liver of mice treated with broad-spectrum antibiotics or vehicle determined by 16S rRNA-Seq. *n* = 5/group. ***P < 0.001. (**F**) Taxonomic composition of microbiota assigned to phylum level in the liver of mice treated with selective antibiotics or vehicle determined by 16S rRNA-Seq. *n* = 5/group. *P < 0.05; **P < 0.01; ***P < 0.001; ****P < 0.0001. (**G**) Weighted PCoA plots of hepatic microbial communities based on Bray-Curtis dissimilarity matrix. Each symbol represents a sample from the liver microbiome of a mouse treated with broad-spectrum or selective antibiotics or vehicle. Clusters were determined by pairwise PERMANOVA. The *x* and *y* axes indicate percentage of variation, and ellipses indicate 95% CI. (**H**) The liver microbiomes in mice treated with broad-spectrum or selective antibiotics were analyzed for α-diversity measures compared with mice treated with vehicle. *n* = 5/group. *P < 0.05; **P < 0.01; ***P < 0.001; ****P < 0.0001.

Since our FMT studies revealed that the liver microbiome is selectively populated from the gut, we postulated that ablation of gut bacteria would reprogram liver microbial communities. Surprisingly, total microbial abundance in the liver did not change with oral antibiotic administration despite a more than 99% decrease in bacterial abundance in the gut (Figure 5, A–C). Nevertheless, oral antibiotics altered the composition of the liver microbiome, markedly decreasing the relative abundance of phylum Bacteroidetes and its subtaxa Bacteroidia, Bacteroidales, and S24-7, whereas other taxa were not affected (Figure 5, D and E). Oral antibiotic treatment similarly decreased the relative abundance of Bacteroidetes in the gut (Supplemental Figure 3A).

Surprisingly, selective oral antibiotic treatment with metronidazole, vancomycin, or combination neomycin/ampicillin each resulted in a decrease in liver Bacteroidetes similar to that seen with broad-spectrum antibiotics (Figure 5F). Analysis of the entire taxonomic hierarchy by LEfSe indicated that, similarly to what occurs with broad-spectrum antibiotic treatment, selective antibiotics also reduced the abundance of subtaxa Bacteroidia and S24-7 (Supplemental Figure 3, B–D). However, metronidazole also increased the prevalence of Firmicutes and decreased Verrucomicrobia, whereas vancomycin and neomycin/ampicillin increased the prevalence of Verrucomicrobia in the liver (Figure 5F). Consistent with these observations, PCoA computed using weighted UniFrac distance metrics indicated that hepatic microbial communities were distinct in the vehicle, panantibiotic, and selective antibiotic-treated groups (Figure 5G). α-Diversity measures indicated decreased richness and reduced evenness in liver microbial communities in mice treated with each of the respective antibiotic regimens (Figure 5H). Further, similarly to what occurred in untreated mice (Figure 1F), broad-spectrum or selective oral antibiotic–treated mice exhibited significant differences between liver and gut bacterial communities (Supplemental Figure 3, E–H).

**Figure 6:**
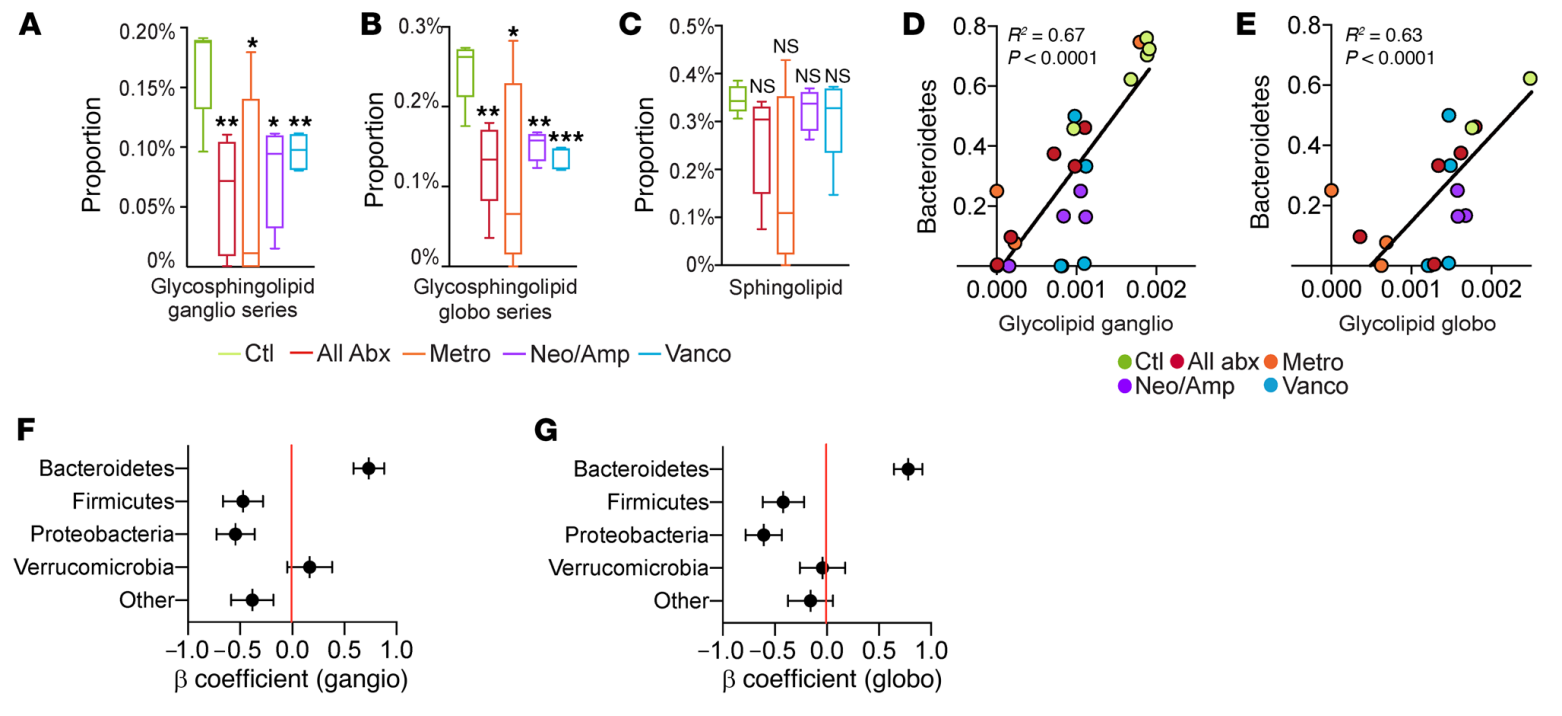
Antibiotic administration alters the glycolipid signature of the liver microbiome. (**A** and **B**) PICRUSt analysis for glycosphingolipid ganglioside (ganglio) (**A**) and globoside (globo) (**B**) biosynthesis pathways in the liver microbiome of mice treated with broad-spectrum or selective antibiotics compared with mice treated with vehicle. *n* = 5/group. *P < 0.05; **P < 0.01; ***P < 0.001. (**C**) PICRUSt analysis for sphingolipid biosynthesis pathways in the liver microbiome of mice treated with broad-spectrum or selective antibiotics compared with mice treated with vehicle. *n* = 5/group. (**D** and **E**) Correlation between glycosphingolipid ganglioside (**D**) and globoside (**E**) biosynthesis and the abundance of Bacteroidetes in the liver microbiome of mice treated with broad-spectrum or selective antibiotics or vehicle. Each colored symbol represents a mouse treated by a specific regimen. (**F** and **G**) Standardized β coefficients for linear regression analysis of the correlation between glycosphingolipid ganglioside (**F**) and globoside (**G**) biosynthesis and the abundance of specific bacterial phyla in the liver of mice treated with broad-spectrum or selective antibiotics or vehicle.

Bacterial taxa in the Bacteroidetes phylum are high producers of glycosphingolipids (10, 11). Based on the markedly reduced prevalence of Bacteroidetes in liver across all antimicrobial treatment groups (Figure 5F), we postulated that glycosphingolipid synthesis by hepatic microbiota would diminish with antibiotic treatment. Accordingly, phylogenetic investigation of communities by reconstruction of unobserved states (PICRUSt)(12) analysis predicted decreased glycosphingolipid biosynthesis in the liver microbiome after treatment with broad-spectrum or selective oral antibiotics, whereas nonglycosylated sphingolipid biosynthesis was not reduced (Figure 6, A–C). Moreover, the predicted contributions of glycosphingolipid biosynthesis genes to the hepatic bacterial metagenome correlated highly with the prevalence of Bacteroidetes in the liver (Figure 6, D and E). Other hepatic bacterial phyla did not exhibit these correlations (Figure 6, F and G). In aggregate, these data indicate that oral antibiotics do not reduce total hepatic bacterial abundance, but selectively modulate the community composition and glycolipid signature of the liver microbiome.

### Sterile liver inflammation reprograms the hepatic microbiome.

We postulated that hepatic injury or sterile inflammation would modulate the liver microbiome. Accordingly, liver microbial populations exhibited distinct clustering based on β-diversity analyses in both chronic liver fibrosis and acute acetaminophen–induced (APAP-induced) liver injury (Supplemental Figure 4, A and B). Chronic liver fibrosis reduced the prevalence of Proteobacteria and Verrucomicrobia, but increased the abundance of Firmicutes, whereas acute liver injury from APAP also reduced the prevalence of Proteobacteria, but increased Verrucomicrobia (Supplemental Figure 4, C and D). Both chronic hepatic fibrosis and acute APAP injury increased richness in the liver microbiome (Supplemental Figure 4, E and F). In contrast, systemic inflammation from i.p. administration of LPS increased the abundance of Bacteroidetes, but reduced Firmicutes, and decreased evenness in the liver microbiome (Supplemental Figure 5, A–C). Collectively, these data indicate that diverse modes of inflammatory injury result in distinct effects on the hepatic microbiome.

### Hepatic inflammatory cell infiltration is dependent on the microbiome.

**Figure 7:**
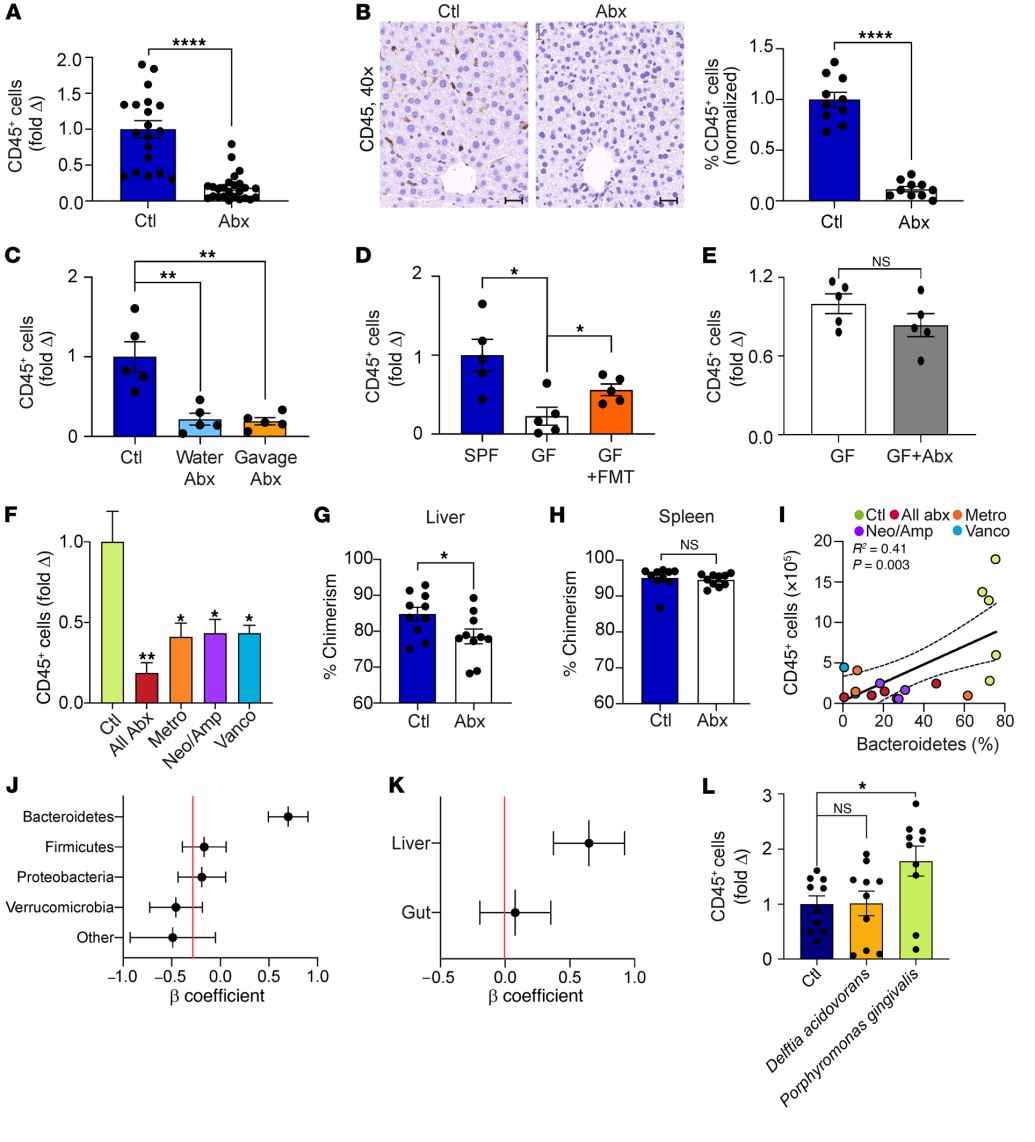
Hepatic immune cell infiltration is contingent on the microbiome. (**A**) Hepatic leukocytes were quantified by flow cytometry in 6-week-old female mice treated with broad-spectrum antibiotics (*n* = 25) or vehicle (*n* = 19).Combined data from 4 experiments are shown. ****P < 0.0001. (**B**) Hepatic leukocytes were quantified as a fraction of nucleated cells by IHC in mice treated with broad-spectrum antibiotics or vehicle. Representative images and quantitative analyses based on 10 HPFs per mouse. *n* = 10/group. ****P < 0.0001. Scale bar = 20µm. (**C**) Hepatic leukocytes were quantified by flow cytometry in control mice or mice treated with broad-spectrum antibiotics added to their drinking water or administered via oral gavage. Data are representative of experiments performed twice. *n* = 5/group. **P < 0.01. (**D**) Hepatic leukocytes in SPF or germ-free (GF) mice that were treated with vehicle or FMT were quantified by flow cytometry. *n* = 5/group. *P < 0.05. (**E**)Hepatic leukocytes in GF mice treated with vehicle or broad-spectrum antibiotics were quantified by flow cytometry . *n* = 5/group. (**F**) Hepatic leukocytes were quantified in mice treated with broad-spectrum or selective antibiotics or vehicle by flow cytometry. Data are representative of experiments performed 3 times. *n* = 5/group. *P < 0.05; **P < 0.01. (**G** and **H**) CD45.2 mice with chimeric CD45.1 bone marrow were treated with oral antibiotics or vehicle starting at week 3. Chimerism in the liver (**G**) and spleen (**H**) was determined at 6 weeks. *n* = 10/group. *P < 0.05. (**I**) Correlation between the number of hepatic immune cells and the abundance of Bacteroidetes in the liver of mice treated with broad-spectrum or selective antibiotics or vehicle. Each colored symbol represents a mouse treated by a specific regimen. (**J**) Standardized β coefficients for simple linear regression analyses of the correlation between the number of hepatic immune cells and the abundance of select phyla in the liver of mice treated with broad-spectrum or selective antibiotics or vehicle. (**K**) Standardized β coefficients for multiple linear regression analysis of the correlation between the number of hepatic immune cells and the abundance of *Bacteroidetes* in the liver and gut of mice treated with broad-spectrum or selective antibiotics or vehicle. (**L**) Six-week-old female mice were treated with broad-spectrum antibiotics and then repopulated with *D*. *acidovorans*, *P*. *gingivalis*, or vehicle by gastric gavage. *n* = 10 mice per group. Hepatic leukocytes were quantified 1 week later by flow cytometry. *P < 0.05.

To determine whether the microbiome influences liver immunity, we serially treated mice with broad-spectrum oral antibiotics for 3 weeks and quantified hepatic immune cells. Antibiotic treatment reduced the total number of CD45^+^ inflammatory cells in the liver by approximately 90% (Figure 7, A and B). A similar reduction in hepatic immune cells was observed regardless of whether antibiotics were administered in the drinking water or via serial gastric gavage (Figure 7C). Accordingly, while livers of germ-free mice harbored a reduced baseline CD45^+^ population, FMT from SPF mice to germ-free mice increased the hepatic leukocyte population (Figure 7D). Of note, treatment of germ-free mice with antibiotics did not affect hepatic inflammatory cell volume (Figure 7E). Likewise, saline gavage did not affect liver leukocyte numbers (Supplemental Figure 6A). We noted that selective antibiotic treatment with metronidazole, vancomycin, or neomycin/ampicillin each similarly reduced the hepatic inflammatory cell population (Figure 7F). We reasoned that the bacteria may control hepatic immune cell volume by affecting both inflammatory cell recruitment to the liver from the bone marrow and intrahepatic leukocyte proliferation. To test the former hypothesis, CD45.2 WT mice were made chimeric with CD45.1 bone marrow cells and, starting at week 3, treated with oral antibiotics or vehicle. We found that antibiotic treatment reduced chimerism in the liver but not the spleen at 6 weeks, indicating that gut microbial ablation prevents hepatic immune cell recruitment from the bone marrow (Figure 7, G and H). Global leukocytic proliferation in the liver was also reduced after antibiotic administration (Supplemental Figure 6B), suggesting that antibiotic-mediated immune cell depletion reflects both decreased recruitment and impaired proliferation.

Based on our observations that both broad-spectrum and selective oral antibiotic regimens had similar effects on diminishing the relative abundance of Bacteroidetes (Figure 5, E and F) and reducing the number of hepatic immune cells (Figure 7F), we postulated that Bacteroidetes drive liver immune cell recruitment. Consistent with our hypothesis, the volume of hepatic immune cells positively correlated with the relative abundance of Bacteroidetes in the liver, which was not the case for other phyla (Figure 7, I and J). Moreover, multiple regression analysis indicated that the prevalence of Bacteroidetes in the liver, but not the gut, was independently associated with hepatic immune cell volume (Figure 7K and Supplemental Table 3). To directly investigate whether Bacteroidetes can promote leukocyte expansion in the liver, we repopulated animals treated with broad-spectrum antibiotics with *P*. *gingivalis*, (a member of *Bacteroidetes* known to produce glycosphingolipids; ref. 13) or *Delftia* *acidovorans* (a member of *Proteobacteria*). Consistent with our hypothesis, repopulation with *P*. *gingivalis* but not *D*. *acidovorans* expanded intrahepatic CD45^+^ inflammatory cells (Figure 7L). Of note, repopulation with *P*. *gingivalis* increased the relative abundance of Bacteroidetes in the liver, but not in the gut (Supplemental Figure 6C).

### The microbiome governs the hepatic immune phenotype.

**Figure 8:**
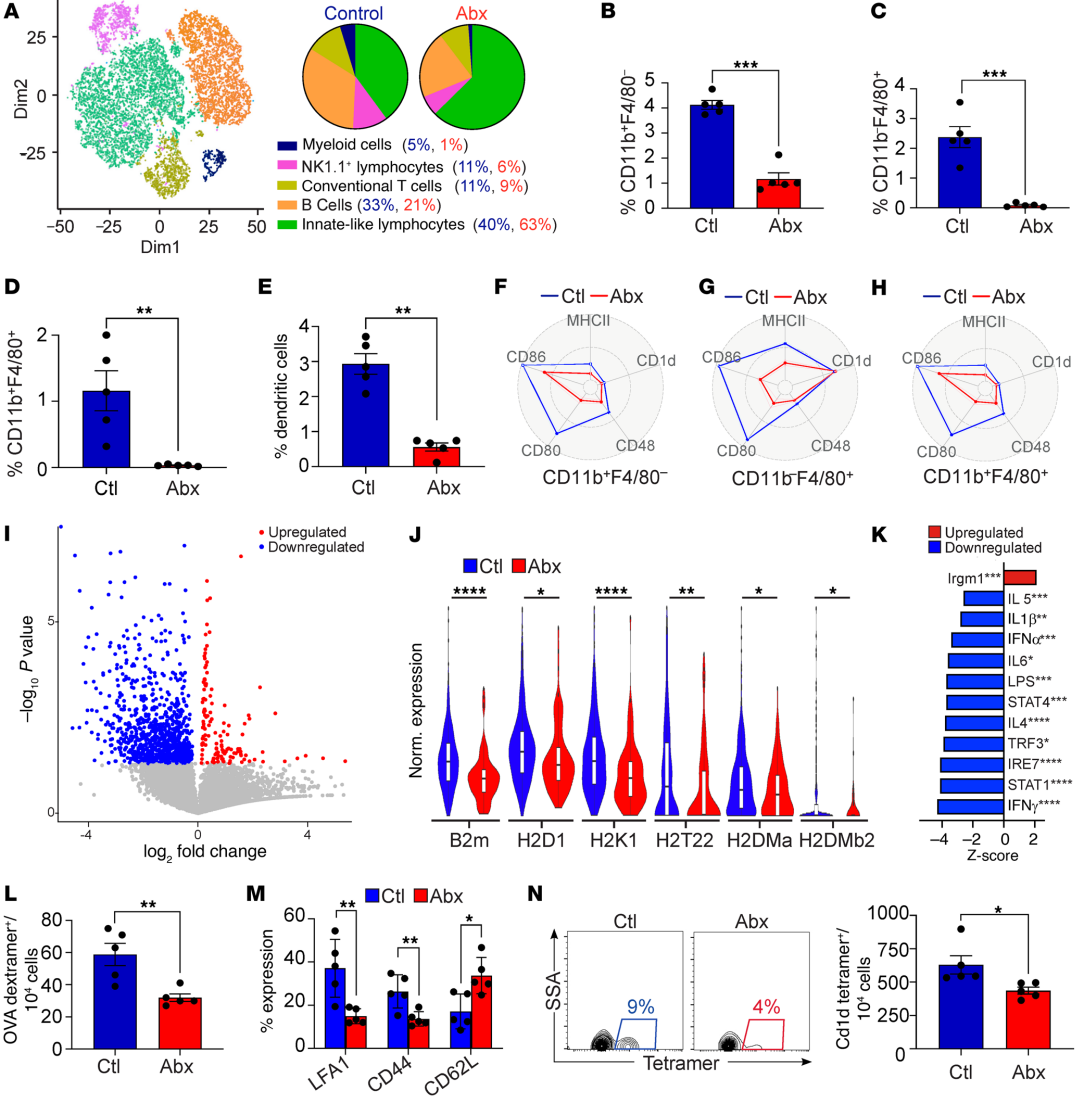
The microbiome promotes the expansion and maturation of liver myeloid cells. (**A**) Six-week-old female mice were treated with either broad-spectrum antibiotics or vehicle. CD45^+^ liver leukocytes were purified by FACS and analyzed by single-cell RNA-Seq. The distribution of cellular clusters was determined using the t-SNE algorithm. Each cluster is identified by a distinct color. Percentage of cellular abundance in each cluster in each respective cohort is depicted in pie charts. (**B**–**E**) The frequency of diverse APC subsets among CD45^+^ liver leukocytes in mice treated with broad-spectrum antibiotics or vehicle was determined by flow cytometry. Data are representative of experiments performed more than 4 times in replicates of 5. **P < 0.01; ***P < 0.001. (**F**–**H**) Expression of activation markers in liver APC subsets in mice treated with broad-spectrum antibiotics or vehicle was determined by flow cytometry and is shown in spider plots. Data are representative of experiments performed more than 4 times in replicates of 5. (**I**) Volcano plot showing differential gene expression in the hepatic myeloid cell cluster for mice treated with broad-spectrum antibiotics versus vehicle based on single-cell RNA-Seq used for **A**. (**J**) Violin plots comparing normalized log expression of select genes in the hepatic myeloid cell cluster for each treatment group shown in **A**. *P < 0.05; **P < 0.01; ****P < 0.0001. (**K**) Changes in upstream regulators in the hepatic myeloid cell cluster for each treatment group shown in **A**. *P < 0.05; **P < 0.01; ***P < 0.001; ****P < 0.0001. (**L** and **M**) Naive mice were immunized twice with OVA-pulsed hepatic macrophages harvested from antibiotic- or vehicle-treated donors. CD8^+^ T cell activation was determined 1 week after the last immunization by measuring the frequency of OVA-dextramer^+^ CTLs (**L**) and their surface phenotype (**M**). This experiment was performed twice. *n* = 5/group. *P < 0.05; **P < 0.01. (**N**) Liver macrophages harvested from antibiotic- or vehicle-treated donors were pulsed with α-GalCer and used to immunize naive mice. The frequency of CD1d-restricted NKT cells was determined 1 week after the second immunization by flow cytometry. This experiment was performed twice. *n* = 5/group. *P < 0.05.

To further characterize the changes in hepatic immunity with antibiotic administration, we performed single-cell RNA-Seq of CD45^+^ hepatic leukocytes in oral antibiotic– and vehicle-treated mice (Figure 8A). Myeloid cells represented 5% of inflammatory cells in control liver, but only 1% in antibiotic-treated mice. Given the approximately 90% reduction of total hepatic leukocytes with antibiotic treatment, the actual volume of myeloid cells was reduced approximately 50-fold after antibiotic administration. The relative abundance of hepatic NK1.1^+^ lymphocytes and B cells was also reduced (Figure 8A). In contrast, the relative frequency of innate-like lymphocytes, including γδT cells, innate lymphoid cells (ILCs), and mucosal-associated invariant T (MAIT) cells was increased. Flow cytometry analysis confirmed a sharp decrease in the frequency of myeloid cells in antibiotic-treated mice, including a reduction in F4/80^–^CD11b^+^, F4/80^+^CD11b^–^, and F4/80^+^CD11b^+^ macrophages and CD11b^–^CD11c^+^MHC II^+^ dendritic cells (Figure 8, B–E). These differences were less pronounced in germ-free mice (Supplemental Figure 6D). Besides depleting these antigen-presenting cell (APC) subsets, oral antibiotic treatment downregulated the expression of MHC II, CD48, CD80, and CD86 (Figure 8, F–H). Of note, most of these changes were not conspicuous in splenic APCs (Supplemental Figure 6, E–G). Similarly, the diminished activation of liver APCs after antibiotic treatment was distinct from the changes seen in intestinal APCs with antibiotic administration (Supplemental Figure 6, H and I). Single-cell RNA-Seq analysis confirmed diminished global activation and reduced expression of antigen presentation machinery in liver myeloid cells after antibiotic treatment (Figure 8, I and J). Further, on ingenuity pathway analysis (IPA), diverse gene sets related to immune response and cellular metabolism were downregulated in hepatic myeloid cells after antibiotic treatment, including oxidative phosphorylation, interferon, chemokine, and iNOS signaling (Supplemental Figure 6J). Examination of upstream regulators confirmed reduced inflammatory signaling in liver myeloid cells after antibiotic treatment (Figure 8K).

Based on these observations, we postulated that hepatic APCs from antibiotic-treated mice would have impaired ability to initiate adaptive immune responses. Accordingly, immunization of naive mice with OVA-pulsed hepatic CD11b^+^ macrophages that were harvested from antibiotic-treated donor mice induced fewer and less activated H-2kb-OVA peptide (SIINFEKL)-dextramer^+^ CTLs compared with immunization with antigen-loaded liver macrophages from control mice (Figure 8, L and M). Likewise, immunization with α-galactosylceramide–pulsed (α-GalCer–pulsed) hepatic macrophages from antibiotic-treated mice induced fewer CD1d-restricted invariant NKT (iNKT) cells compared with controls (Figure 8N). In aggregate, these data indicate that antibiotic treatment diminishes ability of hepatic APCs to activate both conventional T cells and iNKT cells via antigen presentation. Consistent with impaired antigen presentation, hepatic conventional T cells in antibiotic-treated mice exhibited higher expression of the naive T cell markers *Bcl2* and *Il7r* (Supplemental Figure 6K). Nevertheless, single-cell RNA-Seq analysis revealed similar global expression patterns in hepatic conventional T cells in antibiotic-treated and control mice (Supplemental Figure 6, L and M). Akin to our observations in APCs (Supplemental Figure 6, H and I), the antibiotic-associated conventional T cell phenotype in the liver was distinct from that of the gut on the basis of differential changes in the CD8^+^ and CD4^+^FoxP3^+^ T cell populations at each site (Supplemental Figure 6, N and O).

### The microbiome promotes hepatic inflammatory cell recruitment by driving CCL5 signaling in NKT cells.

**Figure 9:**
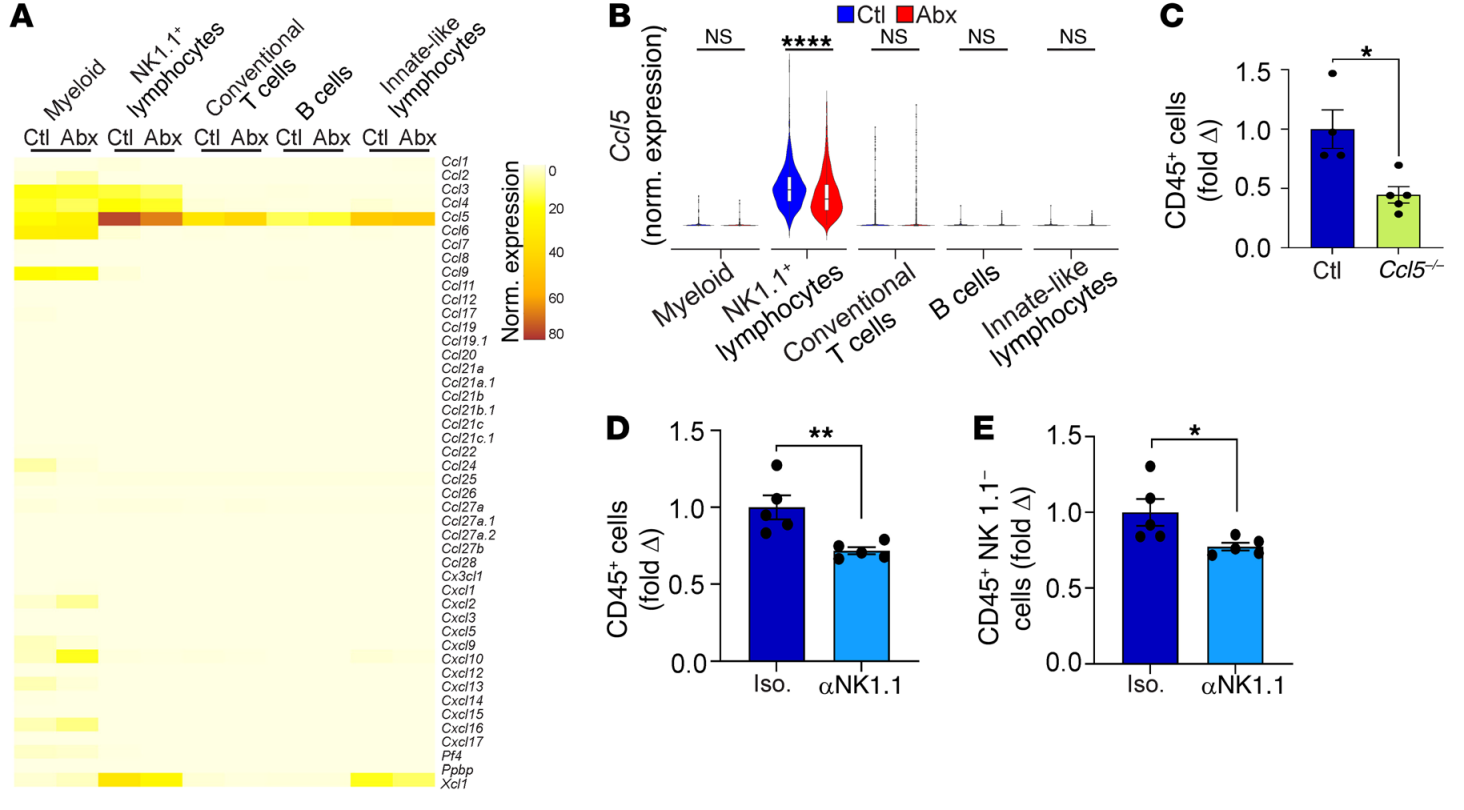
The microbiome drives liver leukocyte recruitment by promoting CCL5 signaling in NK1.1^+^ cells. (**A** and **B**) Mice were treated with broad-spectrum antibiotics or vehicle. CD45^+^ liver leukocytes were purified by FACS and analyzed by single-cell RNA-Seq as in Figure 7A. (**A**) Heatmap depicting expression levels of diverse chemokines in each cluster for both treatment groups. (**B**) Violin plots comparing normalized log expression of *Ccl5* in each cluster for both treatment groups. ****P < 0.0001. (**C**) The total number of hepatic leukocytes was compared in WT versus *Ccl5^–/–^* mice. Data are representative of experiments performed 3 times. *n* = 5/group. *P < 0.05. (**D** and **E**) The number of bulk CD45^+^ (**D**) and CD45^+^NK1.1^–^ (**E**) hepatic leukocytes was compared in WT mice treated with a neutralizing αNK1.1 mAb or isotype control. *n* = 5/group. *P < 0.05; **P < 0.01.

**Figure 10:**
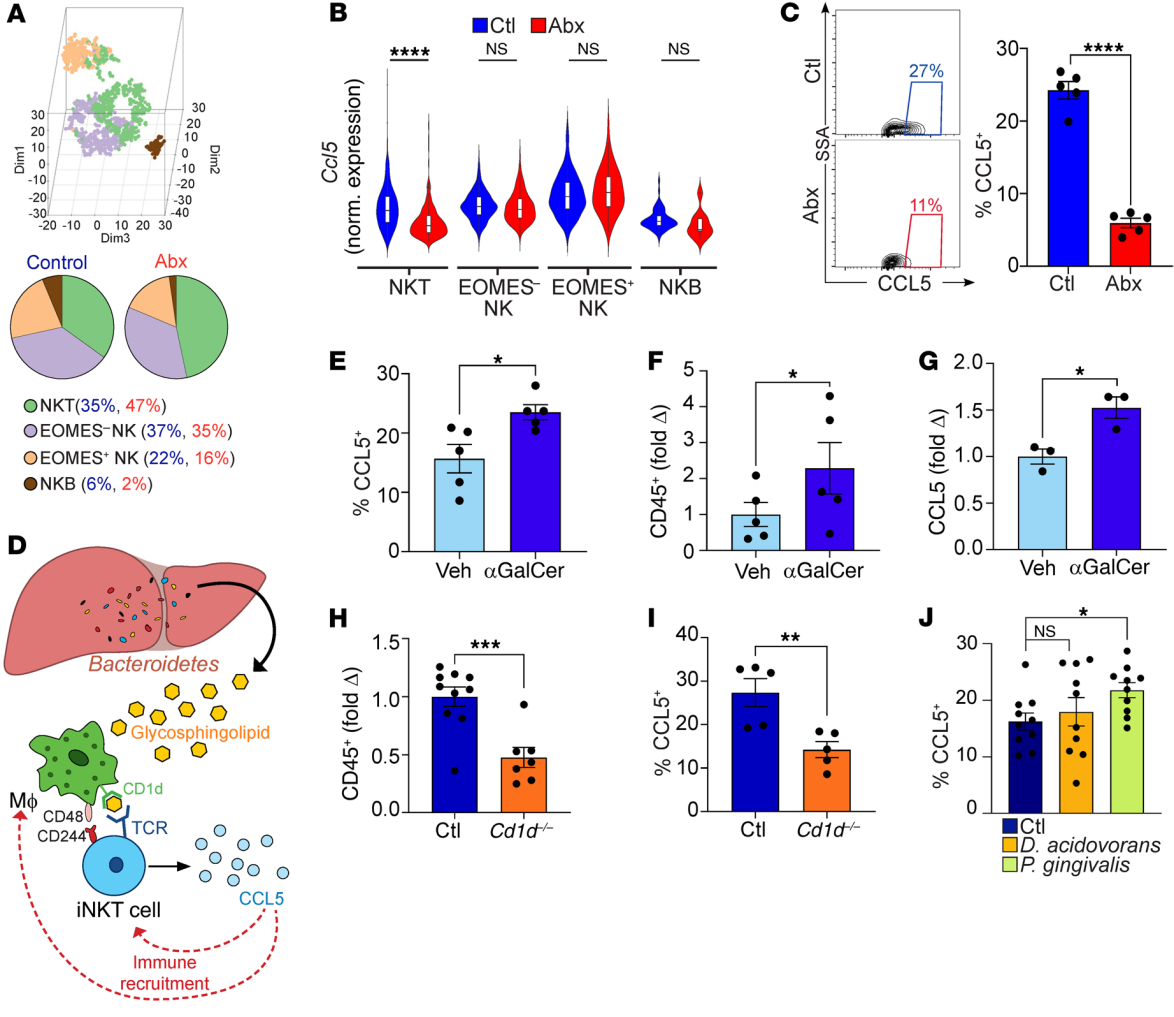
Glycosphingolipid antigens induce CCL5 expression by NKT cells. (**A** and **B**) Mice were treated with broad-spectrum antibiotics or vehicle. CD45^+^ liver leukocytes were purified by FACS and analyzed by single-cell RNA-Seq as in Figure 7A. (**A**) The NK1.1^+^ lymphocyte population was subclustered as shown in a 3D t-SNE plot and quantified in pie charts. (**B**) Violin plots comparing normalized log expression of *Ccl5* in each subcluster for both treatment groups. ****P < 0.0001. (**C**) Mice were treated with broad-spectrum antibiotics or vehicle. Liver NKT cells were analyzed for CCL5 expression. Representative contour plots and quantitative analyses are shown. Data are representative of experiments performed more than 4 times. *n* = 5/group. ****P < 0.0001. (**D**) Schematic of hypothesis that Bacteroidetes-derived glycosphingolipids are presented by APC to iNKT cells, which upregulate CCL5 to potentate leukocyte recruitment. (**E** and **F**) Germ-free WT mice were administered α-GalCer or vehicle i.p. and comparatively analyzed for the frequency of CCL5^+^ NKT cells (**E**) and total CD45^+^ hepatic leukocyte population (**F**). *n* = 5/group. *P < 0.05. (**G**) Hepatic leukocytes were stimulated in vitro with α-GalCer or vehicle, and NKT cells were assayed by flow cytometry for CCL5 expression. Data are representative of experiments performed 3 times in replicates of 5. *P < 0.05. (**H** and **I**) The total number of CD45^+^ hepatic leukocytes (**H**) and CD3^+^NK1.1^+^ cell expression of CCL5 (**I**) were compared in WT and *Cd1d^–/–^* mice. Data are representative of experiments performed 3 times in replicates of 5. **P < 0.01; ***P < 0.001. (**J**) Six-week-old female mice were treated with broad-spectrum antibiotics for and then repopulated with *D*. *acidovorans*, *P*. *gingivalis*, or vehicle by gastric gavage. Liver NKT cells were analyzed 1 week later for expression of CCL5. *n* = 10 mice/group. *P < 0.05.

To investigate the mechanism by which antibiotic treatment reduces the recruitment of hepatic immune cells, we probed our single-cell RNA-Seq data for changes in chemokine expression in the liver upon microbial ablation. *Ccl5* was the most highly expressed chemokine at baseline in nearly all subsets of hepatic leukocytes (Figure 9A). Expression of *Ccl5* was highest in NK1.1^+^ lymphocytes and was markedly reduced with antibiotic administration (Figure 9, A and B). Upstream analysis confirmed that antibiotic administration reduced signaling via CCL5 and its receptors CCR1 and CCR5 in hepatic NK.1.1^+^ cells (Supplemental Figure 7A). Few differences were observed in nonchemokine cytokine expression with antibiotic administration (Supplemental Figure 7B). Based on these data, we postulated that in animals with an intact microbiome, NK1.1^+^ cells recruit hepatic leukocytes via CCL5. Consistent with our hypothesis, *Ccl5^–/–^* mice exhibited a 2-fold reduction in intrahepatic inflammatory cells compared with WT controls (Figure 9C). Similarly, depletion of NK1.1^+^ cells was sufficient to reduce the leukocytic population in the liver (Figure 9, D and E). Of note, livers of *Ccl5^–/–^* mice harbored distinct microbial communities compared with those of WT mice (Supplemental Figure 7, C and D). Subclustering the liver NK1.1^+^ population revealed that NKT cells made up a substantial subset (Figure 10A). Further, NKT cells were the only NK1.1^+^ subpopulation in which *Ccl5* expression was diminished with antibiotic administration, but this decrease was not seen in intestinal NKT cells (Figure 10, B and C, and Supplemental Figure 7E). Further, in contrast with what occurred with conventional T cells (Supplemental Figure 6, L and M), antibiotic administration resulted in global transcriptomic changes in liver NKT cells (Supplemental Figure 7, F and G). Notably, the hepatic CCL5^hi^ NKT cell subset exhibited higher expression of both IFN-γ and IL-10 compared with CCL5^lo^ NKT cells (Supplemental Figure 7H). We noted that, while some cells in the innate-like lymphocyte cluster expressed *Trav11* (the invariant α-chain expressed in murine iNKT cells), these cells did not express high levels of *Ccl5*; moreover, the NKT cell cluster was readily distinguishable on the basis of numerous effector NK markers (Supplemental Figure 7I). Consistent with this, CCL5 was coexpressed with NK1.1 in CD1d-PBS-57 tetramer^+^ iNKT cells (Supplemental Figure 7J).

Broad-spectrum or selective antibiotic treatments each reduced Bacteroidetes abundance in the liver (Figure 5) and diminished microbial production of glycosphingolipids (Figure 6), which are potent CD1d-restricted NKT cell antigens (14). Based on these data, we postulated that activation of hepatic NKT cells by glycosphingolipids drives CCL5 upregulation, promoting liver leukocyte recruitment (Figure 10D). Accordingly, i.p. α-GalCer treatment increased hepatic NKT cell expression of CCL5 and promoted hepatic leukocyte expansion in germ-free mice (Figure 10, E and F). Likewise, in vitro stimulation of hepatic leukocytes with α-GalCer increased CCL5 expression in NKT cells (Figure 10G). Conversely, *CD1d^–/–^* mice, which lack iNKT cells, had fewer hepatic leukocytes and their remaining CD3^+^NK1.1^+^ cell population expressed reduced CCL5 (Figure 10, H and I). Further, consistent with liver-resident Bacteroidetes as drivers of hepatic NKT cell activation, CCL5 expression in NKT cells was increased with *P*. *gingivalis*, but not *D*. *acidovorans*, repopulation (Figure 10J). Collectively, these data suggest that bacterial-derived glycosphingolipid antigens drive CCL5 expression in NKT cells, which potentiate inflammatory cell recruitment to the liver.

Based on TCR sequencing, regardless of treatment, approximately 90% of hepatic NKT cells in C57BL/6 mice were iNKT cells as defined by Vβ2 (*Trbv1*), Vβ7 (*Trbv29*), and Vβ8 (*Trbv13-1*, *Trbv13-2*, *Trbv13-3*) utilization, with Vβ8 the most highly represented (Supplemental Figure 8A). Likewise, nearly all Vβ2^+^, Vβ7^+^, and Vβ8^+^ NKT cells were CD1d-PBS-57 tetramer^+^ (Supplemental Figure 8B). To further characterize the impact of the microbiome on iNKT cell programming, we examined the phenotype of liver iNKT subpopulations after antibiotic treatment. Both the Vβ2^+^ and Vβ8^+^ subsets reduced expression of CCL5 after broad-spectrum antibiotic administration (Supplemental Figure 8C). Further, TCR sequencing indicated that antibiotic administration decreased NKT cell clonal expansion and increased evenness (Supplemental Figure 8D). Taken together, these data suggest that the microbiome governs the hepatic NKT cell phenotype.

### The CD48/CD244 myeloid cell/NKT cell axis drives CCL5 expression.

CD244 is a costimulatory receptor on NKT cells whose ligation by CD48 upon glycosphingolipid presentation by APC accentuates NKT activation (15). We noted that antibiotic administration led to decreased expression of CD244 in hepatic NKT cells (Supplemental Figure 8, E and F). Antibiotic therapy also diminished CD48 expression in diverse liver APC populations (Figure 8, F–H). The CD1d^+^CD48^+^ liver APC subsets were particularly scarce after antibiotic administration (Supplemental Figure 8G). Based on these observations, we postulated that the CD48/CD244 axis between APC and NKT cells potentiates the microbial-dependent upregulation of CCL5 (Figure 10D). Accordingly, CCL5 expression was higher in CD244^+^ NKT cells compared with CD244^–^ NKT cells (Supplemental Figure 8H). Likewise, glycosphingolipids upregulated hepatic NKT cell expression of CD244 in vitro, whereas *Cd1d^–/–^* hepatic NKT cells expressed less CD244 compared with WT (Supplemental Figure 8, I and J). Moreover, repopulation with *P*. *gingivalis*, but not *D*. *acidovorans*, resulted in increased hepatic NKT cell expression of CD244 (Supplemental Figure 8K). Consistent with a receptor-ligand interaction, CD48 blockade in vivo resulted in lower NKT cell expression of both CCL5 and CD244 and reduced the hepatic leukocytic population by approximately 50% (Supplemental Figure 8, L–N). Furthermore, CD1d blockade in vivo inhibited CCL5 expression in CD244^+^, but not CD244^–^, hepatic NKT cells (Supplemental Figure 8O). Collectively, these data indicate that the CD48/CD244 axis potentiates NKT cell expression of CCL5 in response to glycosphingolipid antigens.

## Discussion

The balance between intrahepatic immunity and tolerance is critical to physiologic homeostasis. This is particularly important in the liver, as the portal circulation provides constant exposure to dietary and environmental antigens. Hyperimmunity in the liver is pathogenic in diverse inflammatory and metabolic diseases (16). Conversely, hepatic immune anergy predisposes to infection and primary and metastatic cancer growth (5). We established the presence of bacterial communities within human and murine liver tissues, revealed the dynamic nature of these communities in health and disease, and elucidated their role in leukocyte recruitment and activation. Whereas Proteobacteria are expanded in the liver relative to gut in both mice and humans, we demonstrate that a Bacteroidetes/NKT cell/CCL5 axis is critical for hepatic immune cell expansion and maturation, as glycosphingolipid-producing Bacteroidetes supply CD1d-restricted antigens that drive NKT cell activation and CCL5 expression. Indeed, we found that hepatic NKT cell production of CCL5 and consequent immune cell recruitment to the liver was induced by repopulation of antibiotic-treated mice with a Bacteroidetes species or by introducing the glycosphingolipid α-GalCer into germ-free mice. Moreover, antibiotic treatment not only reduced the overall abundance of microbial-derived glycosphingolipids for NKT activation, but also limited the effectiveness of liver APCs in presenting these and other antigens to adaptive immune cells. The centrality of NKT cells in maintaining hepatic immune competence is consistent with their fundamental role in regulating tumor immunity in the liver, alcoholic and fatty liver diseases, ischemia-reperfusion injury, and autoimmune liver disease (17–22). NKT cells have also previously been shown to regulate APC recruitment to the spleen via CCL5 signaling (23). Here, we found that elevated CCL5 expression was characteristic of hepatic NK1.1^+^ NKT cells, which coexpressed other effector NK markers, but not other *Trav11-*expressing lymphocytes, which likely represent immature NKT cells on the basis of their high *Cxcr6* expression (24). Since expression of *Trav11* is not exclusive to mature NKT cells, and the non-iNKT subset do not express *Trav11*, there is no monoclonal antibody that specifically depletes all NKT cells. Of note, high *CCL5* is also a cluster-defining characteristic of human hepatic NKT cells (25). Moreover, while innate lymphoid populations are abundant in both the liver and the gut, we found that antibiotic treatment decreased CCL5 expression in hepatic, but not gut, NKT cells. We found CCL5 expression in NK1.1^+^ cells, other than NKT cells, suggesting that the axis we describe is not the sole route of CCL5-mediated liver immune-cell recruitment. Notably, the liver microbiome in *Ccl5^–/–^* mice was significantly different from that in WT, suggesting that immune surveillance plays a role in the selection of the liver microbiome, as has previously been shown in the context of mice with impaired innate immunity (26). Importantly, whereas the abundance of Bacteroidetes was increased in the livers of CCL5-deficient mice, the number of immune cells was decreased, consistent with our hypothesis that Bacteroidetes-mediated hepatic immune cell recruitment is CCL5 dependent.

Hepatic abundance of Bacteroidetes was markedly diminished with antibiotic administration, resulting in depletion of liver leukocytes. Likewise, the ability of hepatic APCs to mature and present antigen, including oligopeptides, to conventional T cells or glycosphingolipids to iNKT cells was disrupted by antibiotic administration. Interestingly, the total hepatic bacterial abundance did not change with broad-spectrum antibiotic treatment, which may be related to the increased gut permeability observed with antibiotic treatment (26). The surprising observation that single-agent antibiotic treatment, including with vancomycin, depletes the intrahepatic Bacteroidetes population, may provide insight into the complex mechanisms by which the liver microbiome is selected and may parallel the similar effect of vancomycin on the human and mouse gut microbiome (27–29) either via direct action on Bacteroidetes species, which may be sensitized to vancomycin in vivo due to outer membrane perturbation (30), or by depleting other key microbes. It is plausible that microbial bile acid metabolism, which affects the gut microbiome, has even greater influence on the liver microbiome (31). High levels of secondary bile acids (SBAs) have been shown to promote Bacteroidetes expansion in the gut (32). Conversely, Firmicutes, which are directly targeted by vancomycin, expand in the presence of primary bile acids (PBAs) and drive the 7α-dehydroxylation of PBA to SBA. Whereas depletion of Firmicutes with vancomycin increases PBA (33), FMT can restore SBA production, favoring the restoration of Bacteroidetes in the gut. Hence, to survive in a bile acid–rich environment, Gram-negative Bacteroidetes require the presence of Gram-positive Firmicutes. Additional cooperative and competitive interactions between members of these phyla in the gut have been reported (34). Moreover, given the importance of niche specialization in microbial colonization, it is likely that host-microbe and microbe-microbe interactions shape the liver microbiome in ways that are unique to the hepatic milieu to ensure ecosystem stability. Of note, our PICRUSt analysis was predictive of microbial metabolite changes in the liver, but we did not perform direct metabolomic analyses to precisely characterize these changes. Commensal bacteria in phylum Bacteroidetes are known to produce glycosphingolipids, including CD1d-restricted NKT cell antigens (10, 11, 35). Future work can build on our findings by characterizing the metabolic products of intrahepatic microbes and precisely identifying the Bacteroidetes-derived glycosphingolipids presented to NKT cells in the liver.

The advent of highly sensitive methods of bacterial detection has enabled the characterization of microbiota at sites previously considered sterile (6, 36). For example, we recently described a bacterial community resident in the pancreas that promotes oncogenesis (6). While microbial contamination is of particular concern in the context of a low biomass environment (37), we have employed multiple complementary methods with stringent controls to confirm the presence of bacteria in the liver, including detection of 16S rRNA by qPCR, sequencing, and FISH; electron microscopy; and bacterial culture. Further, our additional findings that physiologic, pathologic, environmental, and pharmacologic perturbations are associated with consistent alterations in the composition of liver microbial communities strongly suggest that these changes reflect real biological phenomena rather than random contamination. Previous evaluation of sampling methods to evaluate murine lung, another low biomass organ, for microbial communities found that whole-tissue sequencing, as we performed in liver, was the superior approach for distinguishing biological signal from contamination (38). Although translocation of specific microbes to the liver has been described in the context of hepatic disease (39–42), as has uptake of blood-borne bacteria by Kupffer cells (43) and *P*. *gingivalis* translocation from the oral cavity to the liver in the setting of periodontal disease (7), a holistic liver microbial community — in the absence of hepatic insult or obstructive cholangiopathy — has not previously been characterized. We noted that microbiota selectively populate the liver in germ-free mice after FMT. The highly selective nature of this process is evident based on the distinct identities of gut versus liver and central versus peripheral hepatic microbial communities, with progressively decreasing richness and evenness over greater anatomic distances from the gut. This is consistent with the branched segmental distribution of the biliary tree and its interstitium and of the portal venous circulation, which communicate between the gut and the liver and represent plausible modes of translocation from the gut to the liver (44). More specifically, there is disproportionate representation of Proteobacteria, and particularly the genus *Delftia*, in the liver microbiome compared with gut. We previously found that these same taxa are also more prevalent in the pancreas than in the gut (6), and here we show that the liver and pancreatic tissue microbiomes cluster closely, perhaps relating to their similar respective tissue host environments. However, the decreased prevalence of Firmicutes in liver compared with gut was not paralleled in the pancreas, which may be a consequence of distinct metabolic or inflammatory niches within each respective organ. The mechanisms of bacterial recruitment and selection within particular organ niches is a rich area for future investigation. Of note, the host may gain evolutionary benefit from limiting Firmicutes colonization of the liver, as SBA, which are produced by Firmicutes, are strongly hepatotoxic (45). However, the broader mechanisms of selection that shape the liver microbiome, which likely include host-immune surveillance and metabolic factors as well as microbial cooperation and competition, require further exploration.

We found that the hepatic microbiome is dynamic. The microbiome in the liver of younger mice, similar to the gut, exhibited a relatively higher prevalence of Bacteroidetes and Firmicutes. In contrast, the liver microbiome of older mice was dominated by Proteobacteria, suggesting that the divergence between the gut and liver microbial communities becomes more prominent with age. Notably, age-related changes in the gut microbiome were not as pronounced as in liver. Gut bacterial dysbiosis has been noted in numerous human liver diseases and in animal models of chronic liver disease (46). Our observation that the liver microbiome changes with acute or chronic hepatic injury raises the possibility that gut microbial derangements may be surrogates for changes in the liver microbiome or that both gut and liver microbes contribute to disease pathogenesis. There are numerous hepatic conditions that are ameliorated with antibiotic therapy, notably hepatocellular carcinoma (47, 48), liver metastases (17, 49), reperfusion injury (50), alcoholic liver disease (51, 52), nonalcoholic steatohepatitis (NASH) (53–56), autoimmune liver disease (57–60), and toxin-induced liver injury (58, 61, 62). Hepatic exposure to microbial metabolites from the intestines has been proposed as a universal mechanism linking dysbiosis to hepatic injury (46), but it is plausible based on our findings that resident liver microbes may also play a role. Of note, high prevalence of Proteobacteria, which include numerous pathogens, is regarded as a marker of dysbiosis in the gut (63), but appears linked to liver homeostasis, as acute or chronic liver injury is associated with decreased prevalence of Proteobacteria in the liver. Our human data were sufficient to confirm the presence of a liver microbiome and the relative enrichment of Proteobacteria compared with what occurred in the gut, but was limited by the number of patients and their diversity of diagnoses and demographics. While we have shown that the liver harbors microbes under normal and pathologic conditions and that these microbes are immunologically significant, their role in the pathogenesis of particular hepatic diseases remains to be explored, particularly via larger prospective trials with appropriately matched cohorts of healthy and diseased human livers. Other questions, such as whether the differences in hepatic immune cell populations between species (e.g., the higher prevalence of NKT cells in mice versus humans) is related to differences in the liver microbiome, will also require larger human data sets. Based on the dynamism of the liver microbiome that we have demonstrated in mice, major differences in hepatic microbial populations are to be expected based on liver or systemic disease, demographic and environmental factors, and dietary intake. We demonstrated the relative stability of orally administered bacteria in the liver compared with the upper gastrointestinal tract over the course of hours to days; the dynamics of the liver microbial community over longer periods of time will require future investigation.

In aggregate, we show that the liver harbors a dynamic microbiome upon which liver immunity is contingent. Further, we establish a pathway linking specific microbial taxa and their metabolites to hepatic immunity. Specifically, we demonstrate that a Bacteroidetes/glycosphingolipid/NKT/CCL5 axis potentiates immune cell recruitment and activation in the liver. This pathway presents an array of therapeutic opportunities for targeting liver microbiota or their downstream effectors in order to modulate a wide variety of liver conditions.

## Methods

### Animals and in vivo models.

C57BL/6, CD45.1, *Ccl5*^–/–^, and *Cd1d*^–/–^ mice were purchased from The Jackson Laboratory. For select experiments, C57BL/6 mice were purchased from Taconic Biosciences. Animals were housed in an SPF barrier vivarium unless otherwise specified and fed standard mouse chow. Germ-free C57BL/6 mice were bred in-house in a gnotobiotic facility. In select experiments, animals were treated i.p. with mAbs targeting NK1.1 (clone PK136), CD48 (clone HM48-1), or CD1d (clone 19G11), 200 μg, all BioXcell or respective isotype controls using regimens we have previously described (64). In other experiments, mice were adoptively transferred with CD45.1 bone marrow cells via retroorbital injection or i.p. with CD11b^+^ cells isolated from liver by positive selection using magnetic beads (Miltenyi Biotec), as we described (65). CD45.2^+^ mice that underwent myeloablation followed by bone marrow reconstitution with CD45.1^+^ donor cells were generated as we described (66). Briefly, animals were irradiated in a SAARP irradiator (Xstrahl Life Sciences) by exposure to 2 fractions of irradiation (5.5 Gy per dose) interspaced by 6 hours, followed by the i.v. transfer of donor bone marrow cells (10^7^ cells) 12 hours after the last dose of radiation. Tissue chimerism was calculated as follows: CD45.1^+^ cells/(CD45.1^+^ cells + CD45.2^+^ cells). To induce hepatic fibrosis, 12-week-old female mice were treated with thrice weekly injections of thioacetamide (TAA) (250 mg/kg; Alfa Aesar for 12 weeks as we previously reported; ref. 67). To induce acute hepatic injury, mice were treated i.p. with 500 μg/g APAP diluted in PBS, as we previously reported (68). To model endotoxemia, mice were injected i.p. with a single dose of LPS, as we previously reported (15 mg/kg; MilliporeSigma) (67). Fecal and tissue specimens were stored in sterile tubes at −80°C until further use.

### Antibiotic treatment and fecal and bacterial transfer experiments.

To ablate the gut microbiome, mice were administered antibiotics in their drinking water for a minimum of 1 week (6). Mouse drinking water was mixed with ampicillin (1 mg/mL; Santa Cruz Biotechnology Inc.), vancomycin (0.5 mg/mL; MilliporeSigma), neomycin (0.5 mg/mL; MilliporeSigma), and metronidazole (1 mg/mL; Santa Cruz Biotechnology Inc.). In select experiments, mice were additionally treated with an oral gavage cocktail containing vancomycin (50 mg/mL; MilliporeSigma), neomycin (10 mg/mL; MilliporeSigma), and metronidazole (100 mg/mL; Santa Cruz Biotechnology Inc.) for 5 days. Control mice were gavaged with PBS. In selective antibiotic experiments, mice received vancomycin only, metronidazole only, or a combination of neomycin and ampicillin at the doses listed above. In fecal transfer experiments, a single mouse fecal pellet was collected and resuspended in 1 mL of PBS, and 200 μL of the fecal slurry was used for orogastric gavage daily for 3 days. Repopulation was confirmed by 16S rRNA-Seq of fecal sample and liver tissues. For bacterial species-specific repopulation experiments, 200 μL of *D*. *acidovorans* (catalog 17438) or *P*. *gingivalis* (catalog 33277, both ATCC; 2 × 10^9^ CFU/mL) with or without fluorescent label (CellTrace Violet, Thermo Fisher) was used to orally gavage mice after gut microbial ablation. Control mice were orally gavaged with PBS.

### Histology and immunohistochemistry.

For histologic analysis, liver specimens were fixed with 10% buffered formalin, dehydrated in ethanol, embedded with paraffin, and stained with H&E or anti-CD45 (catalog ab10558, Abcam), as we previously described (64).

### qPCR.

Total bacterial primers 5′-GTGSTGCAYGGYTGTCGTCA-3′and 5′-ACGTCRTCCMCACCTTCCTC-3′ (69) were used to perform real-time qPCR. The reaction mixture volume was made to a total of 10 μL containing 500 nmol/L of forward and reverse primers, 2× Power SYBR Green Master Mix (Applied Biosystems), and 10 ng sample DNA. The PCR reaction was performed using a Bio-Rad CFX384 real-time system. The reaction cycle was as follows: denaturation at 94°C for 10 minutes, 40 cycles of 94°C for 1 minute, annealing at 60°C for 1 minute, and elongation at 72°C for 90 seconds, followed by a final elongation at 72°C for 5 minutes. The standard curve to calculate bacterial DNA concentration was plotted using *E*. *coli* DNA. To convert Ct values of each sample to total bacterial DNA in the sample, a standard protocol was used (70). The same workflow was used for reagent-only controls.

### FISH.

The EUB338 16S rRNA gene probe labeled with the fluorophore Cy3 (excitation wavelength, 555 nm; emission wavelength, 570 nm; Molecular Probes) was used to detect the bacterial colonization within liver tissues by FISH. In situ hybridization was confirmed using *E*. *coli* (ATCC, catalog PTA-7555) cultured under standard conditions, and fluorescence was compared with the unstained condition. Fluorescence microscopic analysis was conducted with the Nikon Eclipse 90i confocal microscope (Nikon) using a Cy3-labeled probe at 50 pmol/mL, as described (71–73).

### TEM.

Anesthetized mice (C57BL/6) were fixed by cardiac perfusion with freshly prepared solution containing 3% paraformaldehyde in PBS. The liver tissues were dissected, and fixed in 2.5% glutaraldehyde and 2% paraformaldehyde in 0.1M sodium cacodylate buffer (pH 7.2), then post-fixed with 1% osmium tetroxide with 0.8% potassium ferrocyanide in sodium cacodylate buffer for 1.5 hours. The liver tissues were then dehydrated in a graded series of acetone solutions and embedded in EMbed812 epoxy resin (Electron Microscopy Sciences). Ultrathin sections of 70 nm were cut, mounted on copper grids, and stained with uranyl acetate and lead citrate by standard methods. Stained grids were imaged with the Talos120C transmission electron microscope (Thermo Fisher Scientific) using the Gatan OneView digital camera (4K × 4K, Gatan Inc.).

### Human sample collection.

Human liver biopsy specimens were collected in a sterile manner from patients undergoing liver surgery at NYU Grossman School of Medicine. Human fecal samples were collected using rectal swabs. Specimens were stored in sterile TE buffer for 16S sequencing analysis. Patients who had been on antibiotic treatment within the past 3 months or patients who had received neoadjuvant chemotherapy or radiotherapy were excluded. All specimens were stored at −80°C until further use.

### Bacterial DNA extraction and sequencing.

Extraction, quantification, and 16S rRNA-Seq were performed following our routine laboratory procedures (6, 74–76). 16S rRNA library preparation and sequencing were performed using the standard protocol from Illumina (16S metagenomics). 16S rRNA-Seq data are available via the NCBI Sequence Read Archive (SRA PRJNA770739). The GitHub code is available at: https://github.com/mariaasierra/Liver_Microbiome/commits/main (commit ID: 64749b6cd82026cc7099196a9925f6721d6306b6). For details, please refer to Supplemental Methods.

### Statistics.

Data are represented as mean ± SEM. Statistical significance in taxonomic and immune phenotyping studies was determined by Student’s *t* test using GraphPad Prism 8 (GraphPad Software). Simple and multiple linear regression analyses and χ^2^ tests for categorical data were performed using GraphPad Prism 8. *P* < 0.05 was considered significant. Bonferroni’s corrections were performed for analyses of multiple pairwise comparisons.

### Study approval.

Human specimen collection was approved by NYU’s Institutional Review Board and was conducted in accordance with the Declaration of Helsinki, the Belmont Report, and US Common Rule. Donors were deidentified and provided written, informed consent. All animal experiments were approved by the NYU School of Medicine Institutional Animal Care and Use Committee.

## Author contributions

JCL, BP, DS, and GM conceived and planned the experiments. JCL, BP, MMP, SN, CGA, SAAS, MB, IG, LA, YG, AM, MM, ZK, JJP, BH, and AS carried out the experiments. JCL, BP, MMP, SN, CGA, SAAS, MB, IG, LA, YG, MV, ZF, AM, FX, MM, ZK, JJP, and BH contributed to sample preparation. JCL, BP, RC, QL, MMP, SN, MAS, IG, LA, AM, WW, BD, BA, MK, GW, SP, FX, MM, JSP, AS, XL, NDT, DS, and GM contributed to interpretation of the results. JCL, BP, RC, QL, and MAS performed formal analysis. JCL, BP, RC, QL, MAS, SAA, MK, KDM, and FXL contributed to data presentation. FY and ERG carried out the experiments, discussed the results, and commented on the manuscript. All authors discussed the results and commented on the manuscript.

## Supplementary Material

Supplemental data

Supplemental video 1

## Version 1 02/17/2022

In-Press Preview

## Version 2 04/15/2022

Electronic publication
