# Supplementary material for: Intrahepatic microbes govern liver immunity by programming NKT cells
Source: J Clin Invest. 2022 Apr 15;132(8):e151725. doi: 10.1172/JCI151725 (PMC9012289; doi:10.1172/JCI151725)
Supplement: Supplemental data [file jci-132-151725-s159.pdf]

Supplemental Methods

Murine Cellular Isolation, Flow Cytometry, and In Vitro Experiments

Murine hepatic non-parenchymal cells (NPC) were collected as previously described (1). Briefly, the portal vein was cannulated and infused with 1% Collagenase IV (Worthington Biochemical, Lakewood, NJ). The liver was then removed, minced, incubated with Collagenase IV at 37° for 10 minutes, and passed through a 70 μm mesh to obtain a single-cell suspension. Liver non-parenchymal cells were enriched over a 40% Optiprep (Sigma) gradient. Cells were resuspended in ice-cold PBS with 1% FBS. After blocking FcγRIII/II with an anti-CD16/CD32 mAb (eBioscience), cell labeling was performed by incubating 10^6 cells with 1 μg of fluorescently conjugated antibody directed against murine CD45 (30-F11), CD45.1 (A20), CD45.2 (104), CD11b (M1/70), F4/80 (BM8), Gr-1 (RB6-8C5), CD11c (N418), MHC II (M5/114.15.2), CD80 (16-10A1), CD86 (GL-1), CD3 (17A2), TCRβ (H57-597), NK1.1 (PK136), CD48 (HM48-1), CD1d (1B1), CD244 (m2B4[B6]458.1), CCL5 (2E9/CCL5), IL10 (JES5-16E3), IFNγ (XMG1.2), CD4 (RM4-5), CD8a (53-6.7), Vβ2 (B20.6), Vβ7 (TR310; all BioLegend), Vβ8 (REA684; Miltenyi), and FoxP3 (FJK-16s; Invitrogen). Cell preparation for intracellular staining was performed using the Fixation and Permeabilization Solution Kit (eBiosciences). H-2kb-OVA peptide (SIINFEKL)-dextramer staining was performed using an MHC I Dextramer (Immudex, Copenhagen, Denmark). Invariant NKT tetramer staining was performed using the CD1d PBS-57 tetramer (NIH Tetramer Core Facility). Dead cells were excluded from analysis using Zombie Yellow (BioLegend). Flow cytometry was performed on the Attune NxT Acoustic Focusing Cytometer (Thermo Fisher). FACS-sorting was performed on the SY3200 (Sony, Tokyo, Japan). Data were

analyzed using FlowJo (Treestar, Ashland, OR). For hepatic NKT cell stimulation assays, hepatic NPC were cultured with or without α-GalCer (2µg/mL; BioVision, Milpitas, CA).

Single Cell RNA Sequencing

Sequencing results were demultiplexed and converted to FASTQ format using Illumina bcl2fastq software. The Cell Ranger Single-Cell Software Suite (https://support.10xgenomics.com/single-cell-gene-expression/software/pipelines/latest/what-is-cell-ranger) was used to perform sample demultiplexing, barcode processing, and single-cell 3' gene counting. The cDNA insert was aligned to the mm10/GRCm38 reference genome. Only confidently mapped non-PCR duplicates with valid barcodes and UMIs were used to generate the gene-barcode matrix. To account for technical batch differences, we utilized the scSeqR alignment method for data integration. We took the union of the top 2,000 genes with the highest dispersion from both datasets and ran a canonical correlation analysis (CCA) to determine the common sources of variation between datasets. We then aligned the subspaces based of the first 16 canonical correlation vectors, generating a new dimensionality reduction that was then used for further analysis. The data was visualized with t-distributed Stochastic Neighbor Embedding (tSNE) based on the aligned CCA. Marker genes were determined based on differential expression analysis using Wilcoxon rank sum test for each cluster. Cell type identities based on known population markers were assigned as follows: Myeloid cells ($Lyz2^{hi}Apoe^{hi}Lgals3^{hi}Bst2^{hi}Pld4^{hi}Cst3^{hi}$), NK1.1^{+} lymphocytes ($Gzma^{hi}Gzmb^{hi}Ncr1^{hi}Fcer1g^{hi}Klre1^{hi}Klrc2^{hi}$), Conventional T cells ($Cd8b1^{hi}Tcf7^{hi}Lef1^{hi}S1pr1^{hi}Ccr7^{hi}Trac^{hi}$ $Tcrg.C1^{lo}Tcrg.C2^{lo}$), B cells ($CD79a^{hi}CD79b^{hi}Ebf1^{hi}Ighm^{hi}Igkc^{hi}Iglc1^{hi}Iglc2^{hi}Ly6d^{hi}$), Innate-like lymphocytes

(*Cxcr6*^{hi}*Rora*^{hi} *Socs2*^{hi}*Podnl1*^{hi}*Bcl2a1d*^{hi}*Tcrg.C1*^{hi}*Tcrg.C2*^{hi}). For sub-clustering of the NK1.1^{+} lymphocyte cluster, cell type identities were assigned as follows: NKT cells (*Cd3d*^{hi}*Cd3e*^{hi}*Cd8a*^{hi}*Trac*^{hi}*Trdc*^{hi}*Thy1*^{hi}) EOMES^{-} NK cells (*Eomes*^{lo}*Gzmc*^{hi}*Klrb1b*^{hi}*Lag3*^{hi}*Cd200r1*^{hi}*Cd200r2*^{hi}) EOMES^{+} NK cells (*Eomes*^{hi}*Prf1*^{hi}*Klrg1*^{hi}*Klra4*^{hi}*Klra8*^{hi}*Zeb2*^{hi}) NKB cells (*Cd19*^{hi}*CD74*^{hi}*CD79a*^{hi}*Ly6d*^{hi}*Igkc*^{hi}*Iglc2*^{hi}*Iglc3*^{hi}).

T-cell Receptor Sequencing

NK1.1^{+}TCRβ^{+} cells were isolated from hepatic non-parenchymal cells by FACS, and genomic DNA was extracted using DNAeasy mini kit (Qiagen). Mouse TCR sequencing was performed using the immunoSEQ Assay (Adaptive Biotechnologies). V, D, and J segments of the TCR were identified by multiplex PCR using forward primers in each V segment and reverse primers in each J segment. Detected template reads were normalized to total DNA content. Assessment of clonality and sequence overlap analyses were performed on immunoSEQ Analyzer 3.0 software (Adaptive Biotechnologies).

Bacterial Culture, DNA Extraction, and 16S rRNA Sequencing

Liver tissue samples were suspended in 500 μL sterile PBS, vortexed for 30 seconds and sonicated for 15 seconds. For bacterial culture, specimens were plated under aerobic or anaerobic conditions for 72 hours on Tryptic Soy Agar with 5% Sheep's Blood (Molecular Toxicology). For DNA extraction, samples were treated overnight with Proteinase K (2.5 μg/mL) at 55°C, as we described previously (2, 3). Total bacterial genomic DNA was purified from tissue and fecal samples using the QIAamp PowerFecal kit (Qiagen). DNA was quantified for concentration and purity initially by NanoDrop 2000 spectrophotometer (Thermo Scientific), and further verified fluorometrically by

Quant-iT PicoGreen assay (Invitrogen) on SpectraMax M5 microplate reader (Molecular Devices), then stored at −20°C until further analysis. For high-throughput 16S rRNA library preparation and sequencing, the V3–V4 hypervariable region of the 16S rRNA gene was amplified from the genomic DNA of murine and human fecal and liver tissue samples according to the Illumina 16S metagenomics protocol (Part #15044223 Rev. B) using two-step AMPure XP amplification (Beckman Coulter). The DNA concentration was adjusted to 10 ng/μL for all analyses. PCR was performed using the primer set 341F (5′-CCTACGGGNGGCWGCAG-3′) and 805R (5′-GACTACHVGGGTATCTAATCC-3′), each with overhang adapter sequences (IDT) using 2× Kapa HiFi Hotstart ReadyMix DNA polymerase (KapaBiosystems). Samples were amplified in duplicate and purified using AMPure XP beads. Amplification was performed at 95°C (3 minutes), with 25 cycles of 95°C (30 seconds), 55°C (30 seconds), 72°C (30 seconds), and final extension of 72°C (5 minutes). Dual indices from Illumina Nextera XT index kits (Illumina) were added to target amplicons in a second PCR using 2× Kapa HiFi Hotstart ReadyMix DNA polymerase. PCR conditions were 95°C (3 minutes), with 8 cycles of 95°C (30 seconds), 55°C (30 seconds), 72°C (30 seconds), and final extension of 72°C (5 minutes). After each PCR cycle, AMPure XP bead–purified libraries were checked for purity by nanodrop, quantified by PicoGreen assay, and size confirmed on agarose gels. Negative controls were included in all sequencing runs. Equimolar amounts of the generated libraries with dual index were combined and quantified fluorometrically. The pooled amplicon library was denatured, diluted, and sequenced on an Illumina MiSeq platform using MiSeq Reagent Kit v3 (600 cycles) and 300-bp paired-end sequencing protocol.

Quality Control

For adequate quality control, we used best practices of previously published studies (4-6). All the samples were collected using the standard sterile technique (Supplemental Video 1). A new set of sterile instruments was used for each animal. We maintained consistency in DNA extraction techniques and reagents throughout. All PCR reagents were periodically checked for environmental contaminants using 16S universal primers. To control for the quality of our sequencing, we used both predetermined mock communities (such as *E. coli*, *Streptococcus mutans*, and *Fusobacterium nucleatum*) and 'negative' (reagent-only) controls, to check background contamination and the rate of sequencing errors. We included both of these controls in each of the sequencing runs. The blanks/no template controls used in each experiment are reagent controls (containing no DNA) that have passed through every stage of library preparation such as Amplicon PCR, Index PCR, and sequencing step along with other DNA samples.

Phylogenetic and Statistical Analyses

The Illumina-generated sample and reagent control sequence data were processed using the quantitative insights into microbial ecology software package (QIIME) v1.8.0 (7, 8). Reads were quality checked with FASTQ (9) and edited using Cutadapt (10) to remove primer sequences and filter sequences to a minimum length of 100 nucleotides. The forward and reverse Illumina reads were joined using the join_paired_ends.py script and joined reads were demultiplexed and quality filtered using the split_libraries_fastq.py script (11). The filtered sequences were clustered into OTUs based on a 97% similarity threshold using UCLUST algorithm with pick_open_reference_otus.py using the GreenGenes database as reference (12). The chimeric sequences were removed by parallel_identify_chimeric_seqs.py and a phylogeny was created with

make_phylogeny.py using default parameters (13). 16S rRNA sequencing data are available via NCBI Sequence Read Archive accession number PRJNA770739. Code is available at https://github.com/mariaasierra/Liver_Microbiome. The microbial relative abundance plots were generated in R using phyloseq (14). α-diversity plots, such as richness estimators (observed OTUs, ACE, and Chao1) and diversity estimators (Shannon index, Simpson index, and PD), were generated using R-phyloseq and vegan. Two-tailed Student t test was also used when two groups were compared. β-diversity PCoA plots were computed between samples by weighted UniFrac distances, and significance was assessed by the Adonis test (PERMANOVA). LEfSe tool was used to identify differentially significant bacterial taxa between the cohorts with the Kruskal–Wallis test (15). Metagenomic analysis of 16S results was performed using the PICRUSt package in Python (16), using a standard workflow (17). The resulting metagenomic predictions were analyzed by the Student t test or simple linear regression using GraphPad Prism 8 (GraphPad Software). P values < 0.05 were considered statistically significant.

Supplemental Figure 1

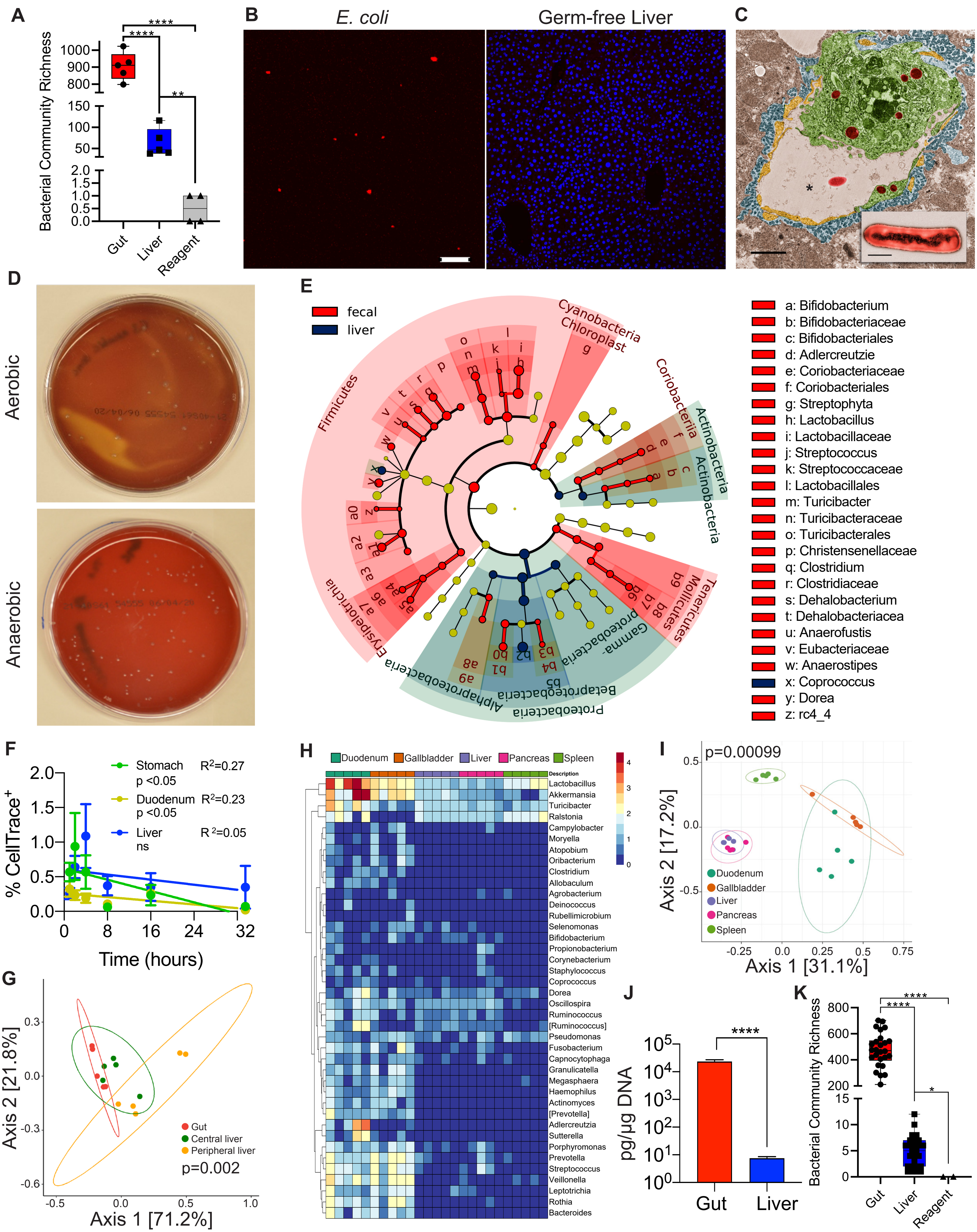

Supplemental Figure Legends

Supplemental Figure 1. The liver and gut microbiomes are distinct.

(A) The liver and gut microbiomes in 6-week-old female WT mice (n=5) and reagent-only controls (n=4) were analyzed for Observed Taxonomic Units (OTUs) (**$p<0.01$, ****$p<0.0001$). Of the reagent-only controls, two had 0 reads past filtering; one had 1 assigned taxon (*Pseudomonas)* and one had 6 unassigned taxa.

(B) *E. coli* were stained using a universal 16S FISH probe vs germ-free mouse liver tissue control (scale bar = 20μm).

(C) Transmission electron microscopy image of mouse liver tissue showing bacteria (red) in relation to endothelial cells (yellow), Kupffer cells (green), the space of Disse (blue), and the sinusoidal space (*, scale bar = 2μm); inset, transmission electron microscopy image of cultured bacteria (scale bar = 1μm).

(D) Mouse liver tissue cultured on tryptic soy agar with 5% sheep's blood plates for 72 hours showing colony growth under aerobic and anaerobic conditions.

(E) Cladogram based on 16S rRNA sequencing of liver and gut microbiomes in 6-week-old female WT mice showing the significant differential bacterial abundances across the entire taxonomic hierarchy in the liver (blue) and gut (red) detected by LEfSe (n=10).

(F) Time course showing the prevalence of fluorescent-labeled *P. gingivalis* in stomach contents, duodenal contents, or liver tissue identified by flow cytometry, with linear regression analysis from 1 hour to 32 hours (n=3 mice / time point).

(G) Weighted PCoA plots based on Bray-Curtis dissimilarity matrix showing distinct clusters for gut microbiota and liver microbiota collected from the central or peripheral

liver. Clusters were determined by pairwise PERMANOVA. X- and Y-axes indicate percent variation and the ellipses indicate 95% CI.

(H) Heatmap showing log2-transformed relative abundances of the most highly represented bacterial genera in duodenum, gallbladder, liver, pancreas, and spleen.

(I) Weighted PCoA plots based on Bray-Curtis dissimilarity matrix showing clusters for microbiota collected from duodenum, gallbladder, liver, pancreas, and spleen. Clusters were determined by pairwise PERMANOVA. X- and Y-axes indicate percent variation and the ellipses indicate 95% CI.

(J) Bacterial DNA content was measured in human gut and liver using qPCR (n=26; ****p<0.0001).

(K) The human liver and gut microbiomes (n=26) and reagent-only controls (n=2) were analyzed for Observed Taxonomic Units (OTUs) (*p<0.05, ****p<0.0001). Both of the reagent-only controls had 0 reads past filtering.

Supplemental Figure 2

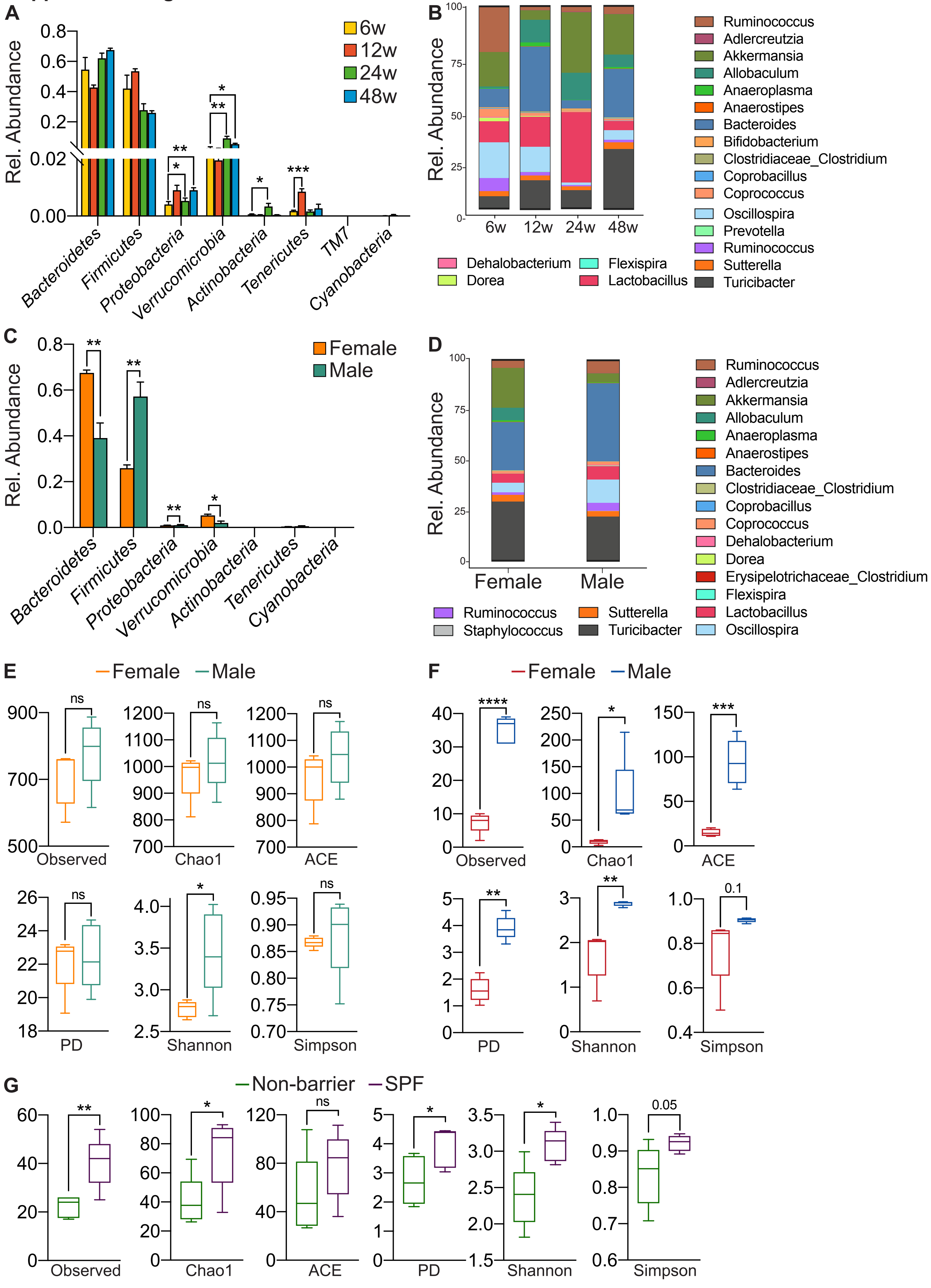

Supplemental Figure 2. Fluctuations in the microbiome based on age, gender, and environment.

(A, B) We comparatively analyzed the gut microbiome in female mice aged 6, 12, 24, or 48 weeks by 16S rRNA sequencing. Taxonomic composition of microbiota in the gut were assigned to phylum (A) and genus (B) levels based on average percent relative abundance (n=5/group).

(C, D) We comparatively analyzed the gut microbiome in female and male mice aged 48 weeks by 16S rRNA sequencing. Taxonomic composition of microbiota in the gut were assigned to phylum (C) and genus (D) levels based on average percent relative abundance (n=5/group).

(E, F) The gut (E) and liver (F) microbiomes in female and male mice were analyzed for α-diversity measures including Observed OTUs, Chao1, ACE, PD, Shannon, and Simpson indices (n=5/group; *p<0.05, **p<0.01, ***p<0.001, **** p<0.0001).

(G) We comparatively analyzed the liver microbiome by 16S rRNA sequencing in cohorts of female mice obtained at 6 weeks of age from Jackson Labs and housed for 3 weeks in SPF vs non-barrier facilities. Liver microbiota were analyzed for α-diversity measures (n=5/group; *p<0.05, **p<0.01).

Supplemental Figure 3

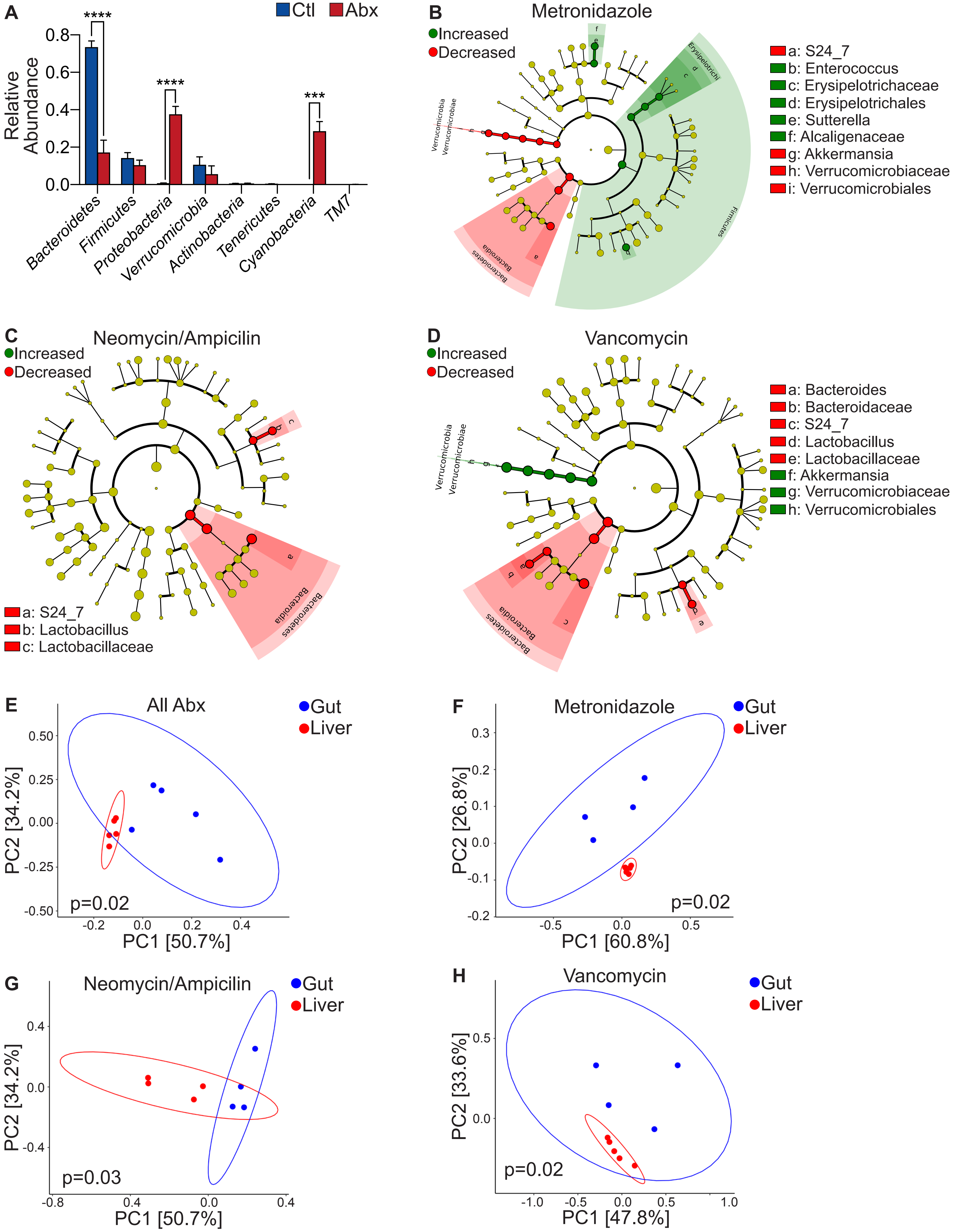

Supplemental Figure 3. Alterations in the liver microbiome with antimicrobial therapy.

(A) Taxonomic composition of microbiota assigned to phylum level in the gut of mice treated with broad-spectrum antibiotics or vehicle determined by 16S rRNA sequencing (n=5/group; ***p<0.001, ****p<0.0001).

(B-D) Cladograms showing significant differential abundances of bacteria in the liver across the entire taxonomic hierarchy detected by LEfSe after treatment of 6-week-old female mice with the indicated selective oral antibiotic regimen vs vehicle.

(E-H) Weighted PCoA plots of gut and liver microbiota in 6-week-old female mice treated with broad-spectrum antibiotics (E) or the indicated selective oral antibiotic regimen (F-H) based on Bray-Curtis dissimilarity matrix. Clusters were determined by pairwise PERMANOVA. X- and Y-axes indicate percent variation and the ellipses indicate 95% CI.

Supplemental Figure 4

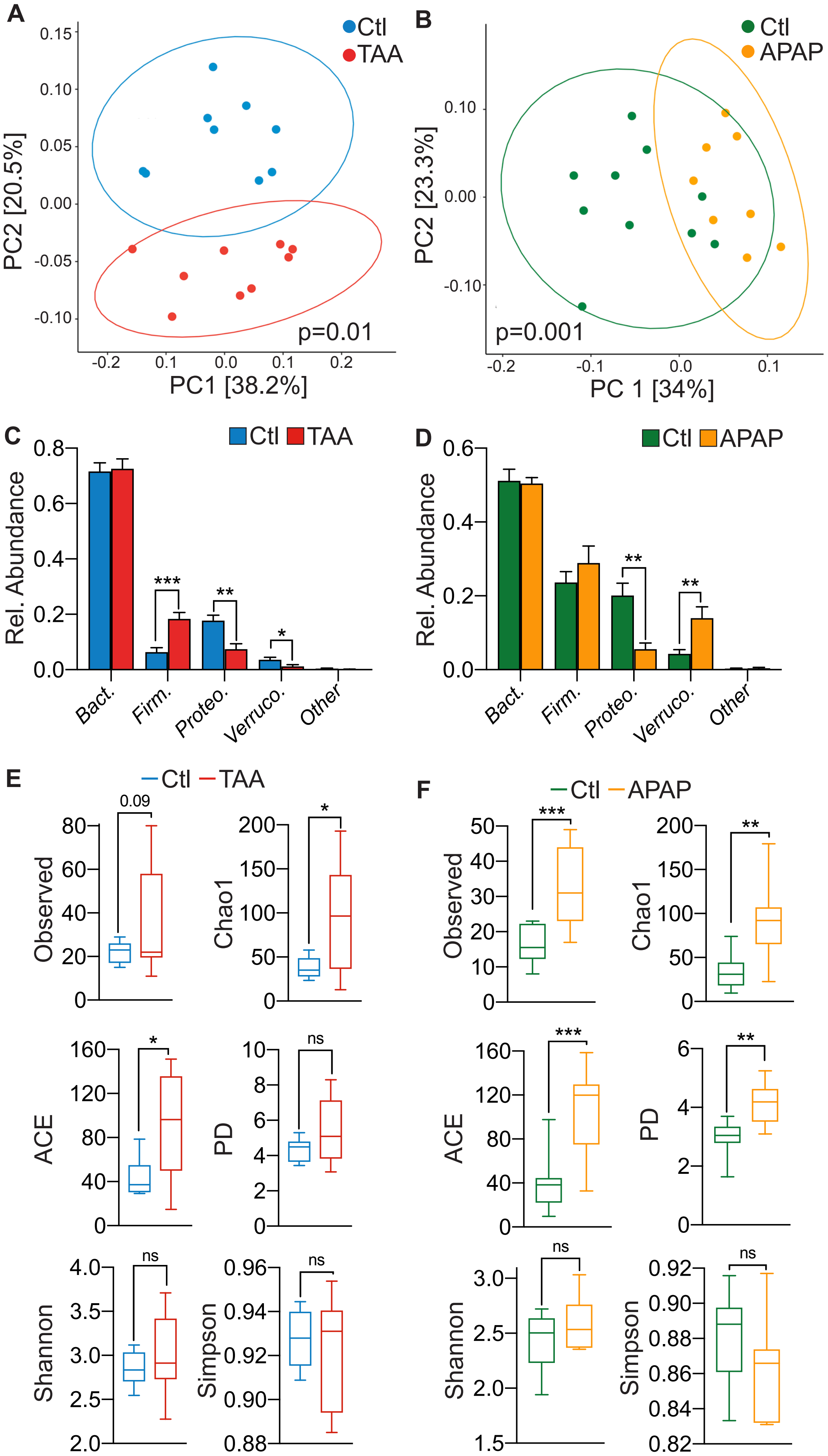

Supplemental Figure 4. Acute or chronic liver disease reprogram the hepatic microbiome.

(A, B) Weighted PCoA plots of liver microbiota in 24-week-old female mice treated with vehicle vs TAA (A) or 6-week-old female mice treated with vehicle vs APAP (B) based on Bray-Curtis dissimilarity matrix. Clusters were determined by pairwise PERMANOVA. X- and Y-axes indicate percent variation and the ellipses indicate 95% CI. Each dot represents data from one mouse.

(C, D) Taxonomic composition of microbiota assigned to phylum level in the liver of 24-week-old female mice treated with vehicle vs TAA (C) or 6-week-old female mice treated with vehicle vs APAP (D) determined by 16S rRNA sequencing (*$p<0.05$, **$p<0.01$, ***$p<0.001$).

(E, F) The liver microbiomes in mice treated with vehicle vs TAA (E) or APAP (F) were analyzed for α-diversity measures including Observed OTUs, Chao1, ACE, PD, Shannon, and Simpson indices (*$p<0.05$, **$p<0.01$, ***$p<0.001$).

Supplemental Figure 5

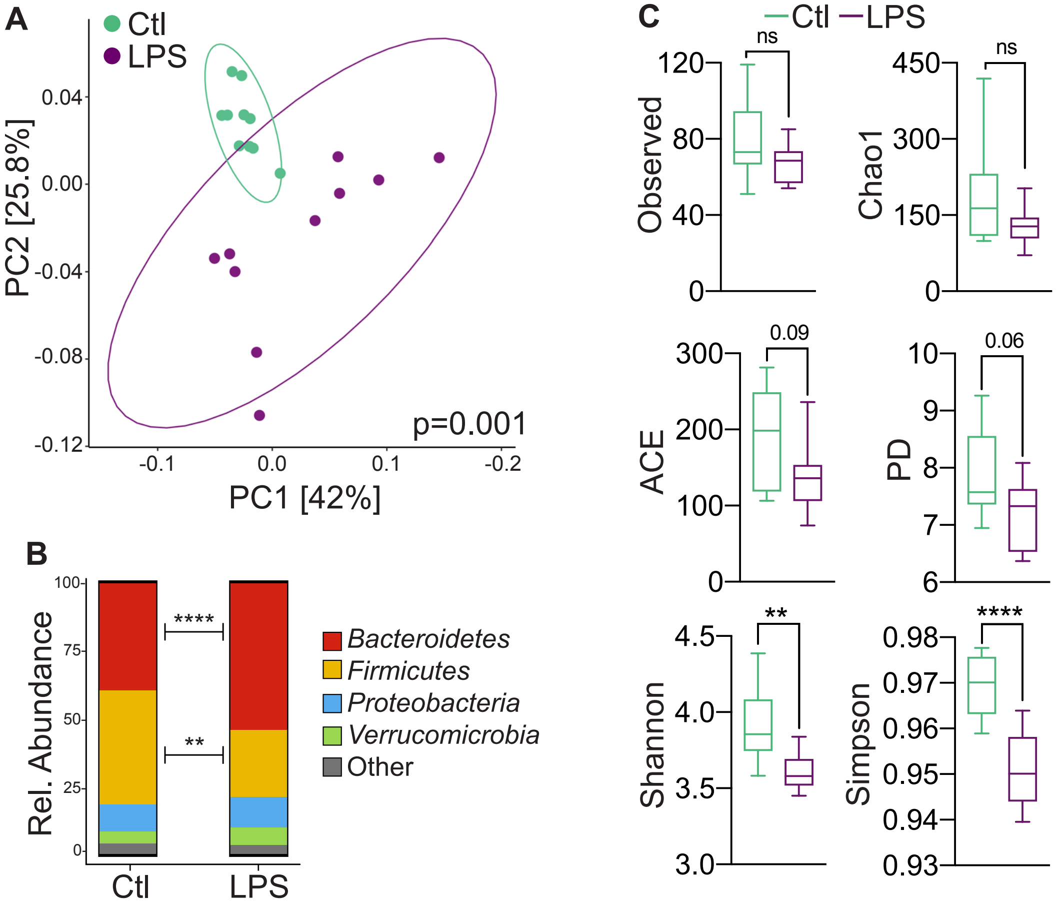

Supplemental Figure 5. LPS administration reprograms the hepatic microbiome.

(A) Weighted PCoA plots of liver microbiota in 6-week-old female mice treated with vehicle vs LPS based on Bray-Curtis dissimilarity matrix. Clusters were determined by pairwise PERMANOVA. X- and Y-axes indicate percent variation and the ellipses indicate 95% CI. Each dot represents data from one mouse.

(B) Taxonomic composition of microbiota assigned to phylum level in the liver of 6-week-old female mice treated with vehicle vs LPS determined by 16S rRNA sequencing (** $p<0.01$, **** $p<0.0001$).

(C) The liver microbiomes in mice treated with vehicle vs LPS were analyzed for α-diversity measures including Observed OTUs, Chao1, ACE, PD, Shannon, and Simpson indices (** $p<0.01$, **** $p<0.0001$).

Supplemental Figure 6

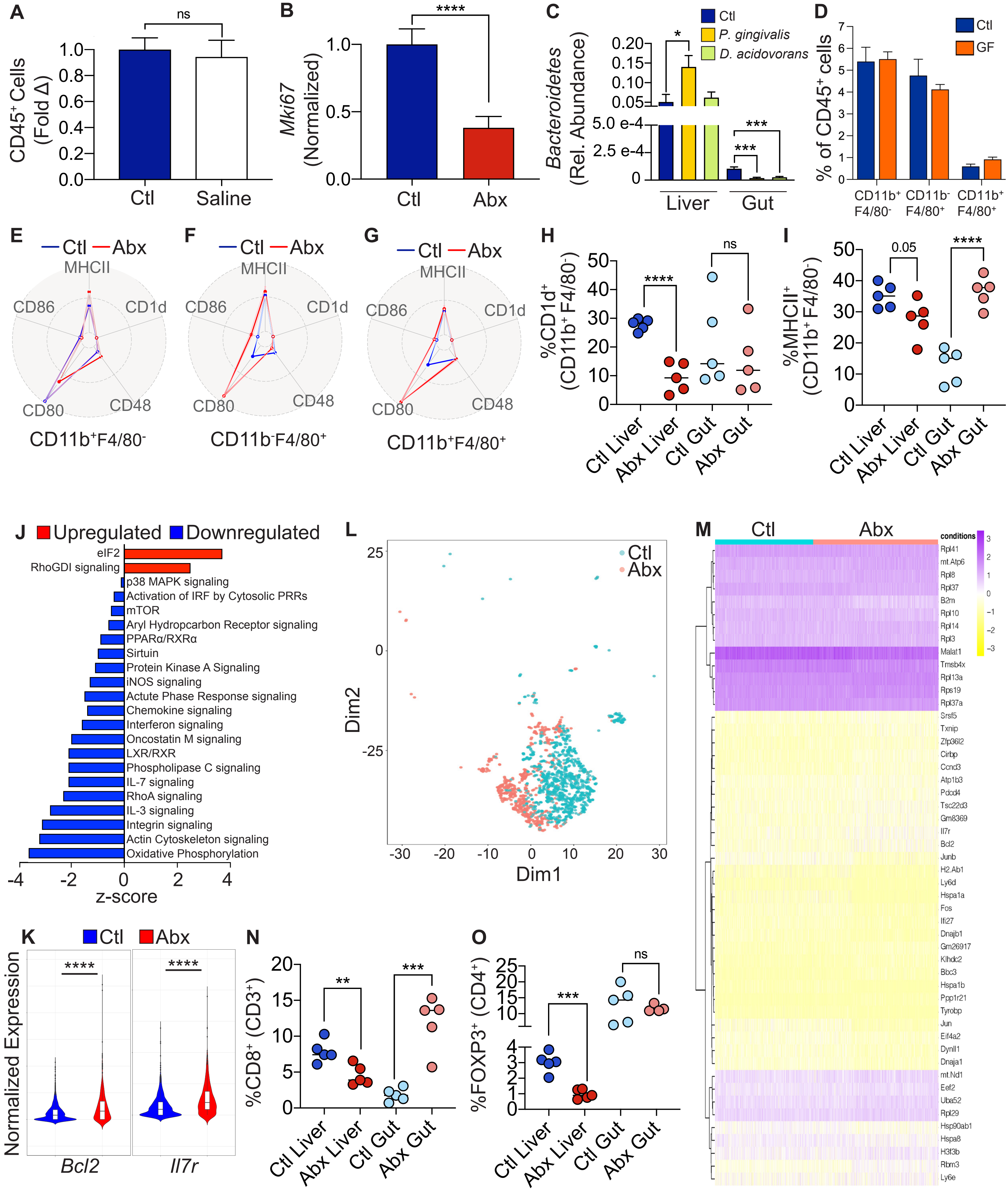

Supplemental Figure 6. Liver myeloid and conventional T cell phenotypes are modulated by antimicrobial therapy.

(A) The number of leukocytes in the liver was comparatively analyzed in mice mock-treated or treated with saline gavage. Data are representative of experiments performed twice in replicates of 5.

(B) Mice were treated with broad-spectrum antibiotics or vehicle. CD45$^+$ liver leukocytes were purified by FACS and analyzed by single cell RNAseq as in Figure 5A. Bar graph comparing normalized log expression of *Mki67* for both treatment groups (****$p<0.0001$).

(C) 6-week-old female mice were treated with broad-spectrum antibiotics for and then repopulated with *D. acidovorans*, *P. gingivalis*, or vehicle by gastric gavage (n=10 mice / group). Relative abundance of phylum *Bacteroidetes* in the liver and the gut was determined by 16S rRNA sequencing (*$p<0.05$, ***$p<0.001$).

(E) The frequency of diverse APC subsets among CD45$^+$ liver leukocytes in control (n=5) and germfree (n=10) mice was determined by flow cytometry (all pairwise comparisons n.s.).

(E-G) Expression of activation markers in spleen APC subsets in mice treated with broad-spectrum antibiotics or vehicle was determined by flow cytometry and is depicted in spider plots. Data are representative of experiments performed twice in replicates of 5.

(H, I) Expression of activation markers in liver and gut APC subsets in mice treated with broad-spectrum antibiotics or vehicle was determined by flow cytometry (n=5/group). This experiment was performed twice.

(J) Mice were treated with broad-spectrum antibiotics or vehicle. $CD45^+$ liver leukocytes were purified by FACS and analyzed by single cell RNAseq as in Figure 5A. The myeloid cell cluster was analyzed by IPA and differentially regulated pathways for antibiotic vs vehicle treatment are shown.

(K-M) Mice were treated with broad-spectrum antibiotics or vehicle. $CD45^+$ liver leukocytes were purified by FACS and analyzed by single cell RNAseq as in Figure 5A. (K) Violin plots comparing normalized log expression of *Bcl2* and *Il7r* in the conventional T cell cluster for both treatment groups (****$p<0.0001$). (L) The respective conventional T cell populations are shown in a t-SNE plot and coded by treatment group. (M) Heatmap showing relative expression of the top 50 differentially expressed genes in the conventional T cell cluster between treatment groups.

(N, O) Frequency of (N) $CD8^+$ T cells and (O) $FOXP3^+$ regulatory T cell subsets in the liver and gut of mice treated with broad-spectrum antibiotics or vehicle were determined by flow cytometry (n=5/group).

Supplemental Figure 7

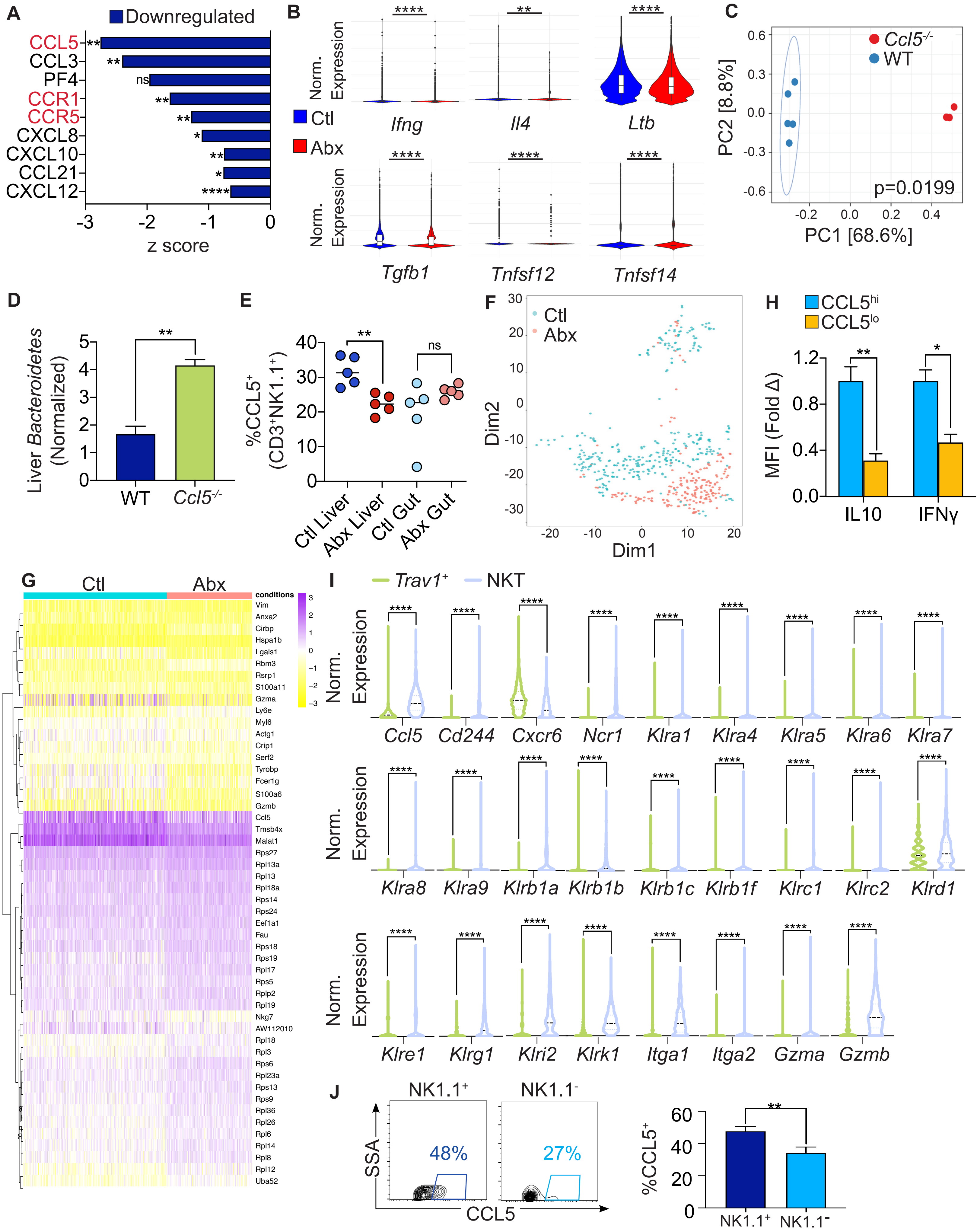

Supplemental Figure 7. The microbiome modulates the phenotype of hepatic NKT cells.

(A) Mice were treated with broad-spectrum antibiotics or vehicle. $CD45^+$ liver leukocytes were purified by FACS and analyzed by single cell RNAseq as in Figure 5A. The $NK1.1^+$ lymphocyte cluster was analyzed by Upstream analysis and differentially regulated chemokine signaling pathways for antibiotic vs vehicle treatment are shown (*p<0.05, **p<0.01, ****p<0.0001).

(B) Mice were treated with broad-spectrum antibiotics or vehicle. $CD45^+$ liver leukocytes were purified by FACS and analyzed by single cell RNAseq as in Figure 5A. Expression of chemokine genes not included in Figure 6A was compared in the overall leukocyte populations. Violin plots comparing normalized log expression of chemokine genes with significant differences for both treatment groups (**, p<0.01, ****p<0.0001).

(C) Weighted PCoA plots based on Bray-Curtis dissimilarity matrix. Each symbol represents a sample from WT (n=5) or *Ccl5*$^{-/-}$ (n=3) liver. Clusters were determined by pairwise PERMANOVA. X- and Y-axes indicate percent variation and ellipses indicate 95% CI.

(D) Relative abundance of phylum *Bacteroidetes* in the livers of WT (n=5) and *Ccl5*$^{-/-}$ (n=3) mice was determined by 16S rRNA sequencing (**p<0.01).

(E) Expression of CCL5 in liver and gut $CD3^+NK1.1^+$ cells in mice treated with broad-spectrum antibiotics or vehicle was determined by flow cytometry (n=5/group). This experiment was performed twice.

(F, G) Mice were treated with broad-spectrum antibiotics or vehicle. $CD45^+$ liver leukocytes were purified by FACS, analyzed by single cell RNAseq as in Figure 5A, and

sub-clustered as in Figure 6F. (F) The respective NKT cell populations are shown in a t-SNE plot and coded by treatment group. (G) Heatmap showing relative expression of the top 50 differentially expressed genes between treatment groups in the NKT cell sub-cluster.

(H) Expression of IL10 and IFNγ in $CCL5^{lo}$ and $CCL5^{hi}$ liver $CD3^{+}NK1.1^{+}$ cells were determined by flow cytometry.

(I) Violin plots comparing normalized log expression of select genes in hepatic innate-like lymphocytes expressing at least one transcript of *Trav11* vs the hepatic NKT cell cluster (****$p<0.0001$).

(J) $NK1.1^{+}$ and $NK1.1^{-}$ liver CD1d-PBS-57 $tetramer^{+}$ cells were comparatively analyzed by flow cytometry for expression of CCL5 (n=5; *$p<0.05$).

Supplemental Figure 8

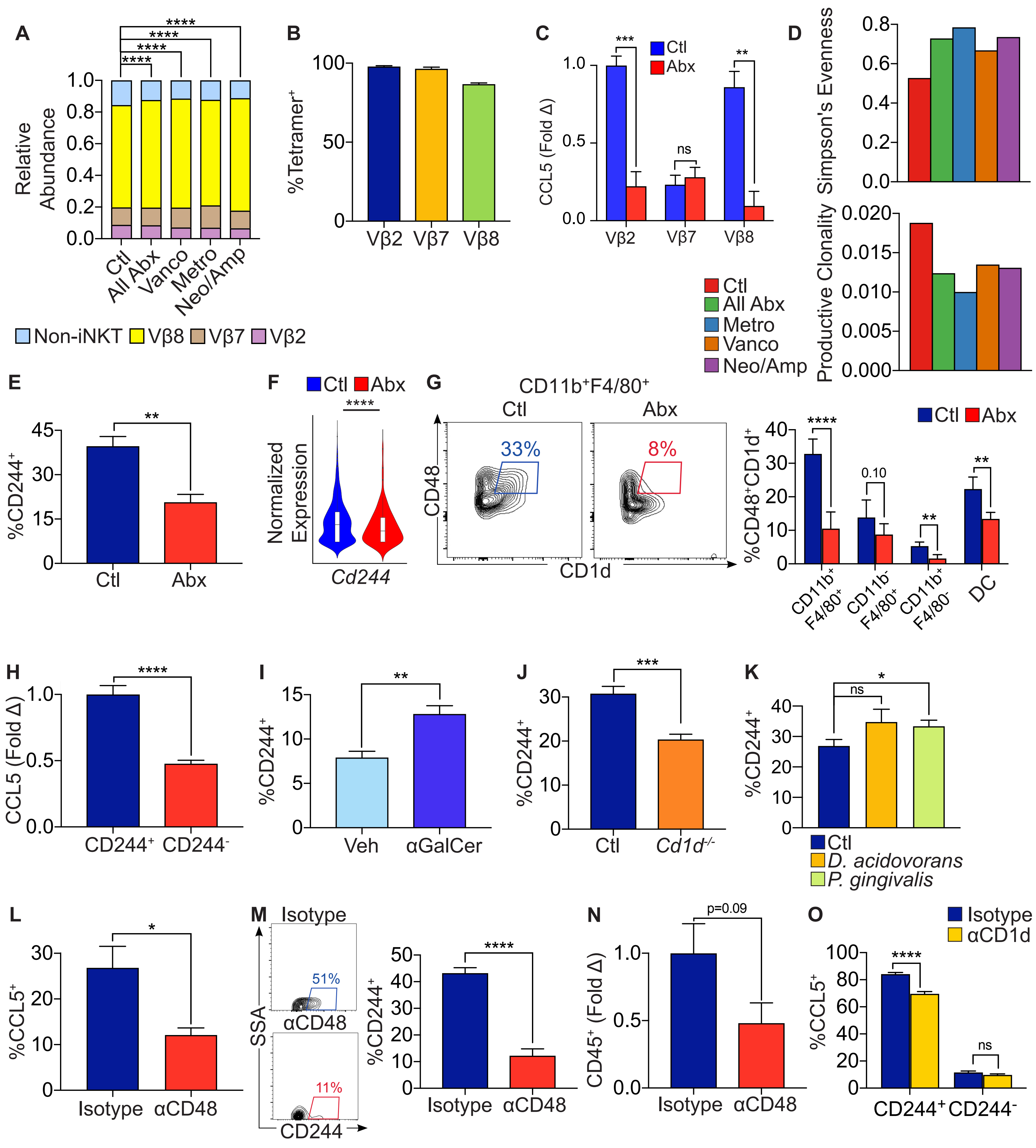

Supplemental Figure 8. The CD48-CD244 axis drives NKT cell activation and CCL5 expression.

(A) TCR sequencing was performed on NKT cells from mice treated with broad-spectrum or selective antibiotics or vehicle. The changes in distribution of iNKT cell subsets is shown and analyzed by chi-square test (****p<0.0001).

(B) Liver iNKT cell subsets were analyzed for CD1d tetramer binding by flow cytometry. The percentage of CD1d tetramer^{+} cells is shown for each iNKT cell subset.

(C) Mice were treated with broad-spectrum antibiotics or vehicle. iNKT cell subsets were analyzed for expression of CCL5 by flow cytometry. Data are representative of experiments performed more than 4 times (n=5 mice/group; **p<0.01, ***p<0.001).

(D) TCR sequencing of NKT cells was performed in mice treated with broad-spectrum or selective antibiotics or vehicle. Productive clonality and Simpson's evenness were determined.

(E) Mice were treated with broad-spectrum antibiotics or vehicle. NKT cell expression of CD244 was determined by flow cytometry. Data are representative of experiments performed 3 times in replicates of 5 (**p<0.01).

(F) Mice were treated with broad-spectrum antibiotics or vehicle. CD45^{+} liver leukocytes were purified by FACS and analyzed by single cell RNAseq as in Figure 5A. Violin plot comparing normalized log expression of *Cd244* in the NK1.1^{+} lymphocyte cluster for both treatment groups (****p<0.0001).

(G) Mice were treated with broad-spectrum antibiotics or vehicle. Liver APC subsets were analyzed for co-expression of CD1d and CD48. Data are representative of experiments performed 3 times in replicates of 5 (**p<0.01, ****p<0.0001).

(H) Liver CD244$^+$ and CD244$^-$ NKT cells were analyzed for expression of CCL5 by flow cytometry. Data are representative of experiments performed 3 times in replicates of 5 (****$p<0.0001$).

(I) Hepatic leukocytes were stimulated *in vitro* with α-GalCer or vehicle and NKT cells were assayed for CD244 expression. Data are representative of experiments performed 3 times in replicates of 5 (**$p<0.01$).

(J) Hepatic NKT cells from WT and *Cd1d*$^{-/-}$ mice were tested for expression of CD244 by flow cytometry. Data are representative of experiments performed 3 times in replicates of 5 (***$p<0.001$).

(K) 6-week-old female mice were treated with broad-spectrum antibiotics for and then repopulated with *D. acidovorans*, *P. gingivalis*, or vehicle by gastric gavage (n=10/group). Liver NKT cells were analyzed 1 week later for expression of CD244 (*$p<0.05$).

(L-N) NKT cell expression of CCL5 (L) and CD244 (M) and total hepatic CD45$^+$ leukocytic population (N) were compared in WT mice treated with a neutralizing αCD48 Ab or isotype control. Data are representative of experiments performed twice (n=5/group; *$p<0.05$, ****$p<0.0001$).

(O) CD244$^+$ and CD244$^-$ NKT cell expression of CCL5 was compared in WT mice treated with a neutralizing αCD1d Ab or isotype control (n=10/group; ****$p<0.0001$).

Supplemental Tables

Table S1. Average threshold cycle (Ct) values for 16S rRNA qPCR.

	**Mouse**	**Human**
**Gut**	15.51	12.16
**Liver**	31.24	25.92
**Reagent control**	36.37	35.70

Table S2. Characteristics of patients in study.

**Patient**	**Age (years)**	**Sex**	**Serum total bilirubin (mg/dL)**	**Diagnosis**
1	57	F	0.5	Benign hepatic cyst
2	52	M	0.7	Chronic cholecystitis
3	71	M	1.0	Hepatocellular carcinoma
4	83	F	0.5	Colorectal adenocarcinoma
5	71	M	0.3	Gastric adenocarcinoma
6	67	M	0.8	Hepatocellular carcinoma
7	56	M	1.8	Hepatocellular carcinoma
8	55	M	0.4	Hepatocellular carcinoma
9	55	M	0.7	Chronic cholecystitis
10	30	M	1.2	Colorectal adenocarcinoma
11	63	M	0.3	Gastric metaplasia
12	37	F	0.5	Ovarian cancer
13	62	F	0.2	Ampullary adenocarcinoma
14	48	F	0.5	Colorectal adenocarcinoma
15	72	M	0.3	Hepatocellular carcinoma
16	55	F	0.6	Benign pancreatic tumor
17	68	F	0.4	Gallbladder cancer
18	61	M	0.5	Hepatocellular carcinoma
19	43	F	0.4	Breast adenocarcinoma
20	50	F	0.9	Breast adenocarcinoma
21	49	F	0.7	Benign hepatic adenoma
22	76	F	0.5	Cholangiocarcinoma
23	51	M	1.0	Hepatocellular carcinoma
24	77	M	0.8	Hepatocellular carcinoma
25	61	M	0.7	Hepatocellular carcinoma
26	84	M	1.0	Hepatocellular carcinoma

Table S3. Liver *Bacteroidetes* independently correlates with hepatic immune cell volume*.

Variable	Parameter estimate	Standard error	95% CI	|t|	P value
Intercept	17550	153030	-306859 – 341959	0.1147	0.9101
% *Bacteroidetes* (Liver)	1045913	443852	104990 – 1986836	2.356	0.0315
% *Bacteroidetes* (Gut)	110709	384726	-704873 – 926291	0.2878	0.7772

*Multiple regression analysis with total hepatic immune cells as outcome variable.

Supplemental References

1. Torres-Hernandez A, Wang W, Nikiforov Y, Tejada K, Torres L, Kalabin A, et al. Targeting SYK signaling in myeloid cells protects against liver fibrosis and hepatocarcinogenesis. *Oncogene.* 2019;38(23):4512-26.
2. Pushalkar S, Ji X, Li Y, Estilo C, Yegnanarayana R, Singh B, et al. Comparison of oral microbiota in tumor and non-tumor tissues of patients with oral squamous cell carcinoma. *BMC Microbiol.* 2012;12:144.
3. Pushalkar S, Li X, Kurago Z, Ramanathapuram LV, Matsumura S, Fleisher KE, et al. Oral microbiota and host innate immune response in bisphosphonate-related osteonecrosis of the jaw. *Int J Oral Sci.* 2014;6(4):219-26.
4. Goodrich JK, Di Rienzi SC, Poole AC, Koren O, Walters WA, Caporaso JG, et al. Conducting a microbiome study. *Cell.* 2014;158(2):250-62.
5. Mukherjee S, Huntemann M, Ivanova N, Kyrpides NC, and Pati A. Large-scale contamination of microbial isolate genomes by Illumina PhiX control. *Stand Genomic Sci.* 2015;10:18.
6. Sinha R, Abnet CC, White O, Knight R, and Huttenhower C. The microbiome quality control project: baseline study design and future directions. *Genome Biol.* 2015;16:276.
7. Caporaso JG, Lauber CL, Walters WA, Berg-Lyons D, Huntley J, Fierer N, et al. Ultra-high-throughput microbial community analysis on the Illumina HiSeq and MiSeq platforms. *ISME J.* 2012;6(8):1621-4.
8. Navas-Molina JA, Peralta-Sanchez JM, Gonzalez A, McMurdie PJ, Vazquez-Baeza Y, Xu Z, et al. Advancing our understanding of the human microbiome using QIIME. *Methods Enzymol.* 2013;531:371-444.
9. Brown J, Pirrung M, and McCue LA. FQC Dashboard: integrates FastQC results into a web-based, interactive, and extensible FASTQ quality control tool. *Bioinformatics.* 2017.
10. Kechin A, Boyarskikh U, Kel A, and Filipenko M. cutPrimers: A New Tool for Accurate Cutting of Primers from Reads of Targeted Next Generation Sequencing. *J Comput Biol.* 2017;24(11):1138-43.

11. Caporaso JG, Kuczynski J, Stombaugh J, Bittinger K, Bushman FD, Costello EK, et al. QIIME allows analysis of high-throughput community sequencing data. *Nat Methods.* 2010;7(5):335-6.
12. Edgar RC. Search and clustering orders of magnitude faster than BLAST. *Bioinformatics.* 2010;26(19):2460-1.
13. Price MN, Dehal PS, and Arkin AP. FastTree: computing large minimum evolution trees with profiles instead of a distance matrix. *Mol Biol Evol.* 2009;26(7):1641-50.
14. McMurdie PJ, and Holmes S. phyloseq: an R package for reproducible interactive analysis and graphics of microbiome census data. *PLoS One.* 2013;8(4):e61217.
15. Segata N, Izard J, Waldron L, Gevers D, Miropolsky L, Garrett WS, et al. Metagenomic biomarker discovery and explanation. *Genome Biol.* 2011;12(6):R60.
16. Langille MG, Zaneveld J, Caporaso JG, McDonald D, Knights D, Reyes JA, et al. Predictive functional profiling of microbial communities using 16S rRNA marker gene sequences. *Nat Biotechnol.* 2013;31(9):814-21.
17. Douglas GM, Beiko RG, and Langille MGI. Predicting the Functional Potential of the Microbiome from Marker Genes Using PICRUSt. *Methods Mol Biol.* 2018;1849:169-77.
